# Mental health literacy measures evaluating knowledge, attitudes and help-seeking: a scoping review

**DOI:** 10.1186/s12888-015-0681-9

**Published:** 2015-11-17

**Authors:** Yifeng Wei, Patrick J. McGrath, Jill Hayden, Stan Kutcher

**Affiliations:** Sun Life Financial Chair in Adolescent Mental Health team, IWK Health Centre, Dalhousie University, 5850 University Ave., P.O Box 9700, Halifax, Nova Scotia B3K 6R8 Canada; IWK Health Centre, Nova Scotia Health Authority and Dalhousie University, 5850 University Ave., P.O Box 9700, Halifax, Nova Scotia B3K 6R8 Canada; Centre for Clinical Research, Dalhousie University, Room 403, 5790 University Avenue, Halifax, Nova Scotia B3H IV7 Canada

**Keywords:** Scoping review, Mental health literacy, Measures, Psychometric properties

## Abstract

**Background:**

Mental health literacy has received increasing attention as a useful strategy to promote early identification of mental disorders, reduce stigma and enhance help-seeking behaviors. However, despite the abundance of research on mental health literacy interventions, there is the absence of evaluations of current available mental health literacy measures and related psychometrics. We conducted a scoping review to bridge the gap.

**Methods:**

We searched PubMed, PsycINFO, Embase, CINAHL, Cochrane Library, and ERIC for relevant studies. We only focused on quantitative studies and English publications, however, we didn’t limit study participants, locations, or publication dates. We excluded non-English studies, and did not check the grey literature (non peer-reviewed publications or documents of any type) and therefore may have missed some eligible measures.

**Results:**

We located 401 studies that include 69 knowledge measures (14 validated), 111 stigma measures (65 validated), and 35 help-seeking related measures (10 validated). Knowledge measures mainly investigated the ability of illness identification, and factual knowledge of mental disorders such as terminology, etiology, diagnosis, prognosis, and consequences. Stigma measures include those focused on stigma against mental illness or the mentally ill; self-stigma ; experienced stigma; and stigma against mental health treatment and help-seeking. Help-seeking measures included those of help-seeking attitudes, intentions to seek help, and actual help-seeking behaviors.

**Conclusions:**

Our review provides a compendium of available mental health literacy measures to facilitate applying existing measures or developing new measures. It also provides a solid database for future research on systematically assessing the quality of the included measures.

**Electronic supplementary material:**

The online version of this article (doi:10.1186/s12888-015-0681-9) contains supplementary material, which is available to authorized users.

## Background

### Epidemiology of mental illness

Approximately 70 %-75 % of adult mental health problems and mental disorders start to manifest during adolescence or early adulthood (12-25) [1, 2]. Globally, mental disorders make up about 1/3 of the burden of illness in adolescence and young adulthood [3]. Untreated mental health problems and disorders in adolescents and young adults are strong predictors of poor vocational achievements, problematic interpersonal and family functioning, as well as reduced life expectancy due to associated medical conditions, such as diabetes, heart diseases and stroke, respiratory conditions, and suicide [4–7]. However, despite the great burden of illness incurred by these conditions, research shows, worldwide, between 70 %-80 % of young people and adults do not receive the mental health care they need [8–10]. A recent systematic review [11] of perceived barriers and facilitators for mental health help-seeking indicated that perceived stigma and embarrassment, problems in symptom identification and a preference for self-reliance were the most important intra-personal barriers to mental health help-seeking.

### Mental health literacy

Mental health literacy is a significant determinant of mental health and has the potential to improve both individual and population health [12–14]. Evidence shows that improved knowledge about mental health and mental disorders, better awareness of how to seek help and treatment, and reduced stigma against mental illness at individual, community and institutional levels may promote early identification of mental disorders, improve mental health outcomes and increase the use of health services [15–17].

We conceptualize mental health literacy to include 4 domains: 1) understanding how to obtain and maintain good mental health; 2) understanding mental disorders and their treatments; 3) decreasing stigma against mental illness; and 4) enhancing help-seeking efficacy [13, 18]. And therefore, mental health literacy addresses 3 inter-related cocepts: knowledge (knowledge of mental illness and positive mental health), attitudes and help-seeking efficacy. This definition is consistent with the current construct of health literacy defined and promoted by the WHO as an empowerment tool for people to participate in their health care [19].

We located five reviews on the effectiveness of mental health literacy interventions [12, 18, 20–22]. In addition there were three literature reviews describing stigma [23–25] and knowledge measures. However, there has been a lack of comprehensive understanding of current available mental health literacy measures. Thus, there exists a need to conduct a study to help better understand strengths and weaknesses of existing measures and to help shape future development of measures. We conducted a scoping review, a systematic approach to map the literature in an area of interest and to accumulate and synthesize evidence available, to bridge the gap. This current scoping review was guided by Arksey and O’Malley’s work (2005) [26], proposing four purposes: 1. to examine the extent, range and nature of research activity; 2. to determine the value of undertaking a full systematic review; 3. to summarize and disseminate research findings; and 4. to identify research gaps in the existing literature.

We analyzed available mental health literacy measures that focus on four common mental disorders with onset before or during adolescence and young adulthood: Schizophrenia, Depression, Anxiety Disorders, and Attention Deficit Hyperactivity Disorder (ADHD).

## Methods

We used our definition of mental health literacy [13, 18] that is composed of 4 constructs addressing three outcomes: mental health knowledge (including knowledge about positive mental health (construct 1) and knowledge about mental illness and treatments (construct 2)), stigma/attitudes towards mental illness, and help-seeking, to define our search scope.

### Search strategy

One of the authors of this review and a health librarian designed the search strategies together. We searched PubMed, PsycINFO, Embase, CINAHL, Cochrane Library, and ERIC between 2013 and 2014, and re-ran the search in 2015. We applied four sets of search terms to identify domains of mental health literacy as outlined in Additional file 1.

### Inclusion criteria

We included quantitative studies that used, developed, or investigated measurement properties of mental health literacy measures evaluating any one, or combinations of the mental health literacy outcomes: knowledge, stigma/attitudes towards mental disorders, and help-seeking. Study designs included any type of quantitative studies: randomized controlled trials (RCTs), cluster RCTs, quasi-experimental studies; cohort studies; cross-sectional/survey studies, and controlled-before-and-after studies (pre/post tests). Only studies published in English were eligible and non-English publications were excluded at the screening stage. Year of publication and study participants, including their age, were not restricted.

### Exclusion criteria

Studies were not eligible if they addressed mental health literacy but did not mention or describe the measure applied in the study. Studies of smoking prevention/cessation and other substance use prevention programs were not included. Studies of suicide prevention interventions that did not address related mental disorders, such as Depression were not eligible. Qualitative studies were excluded.

### Data extraction and study selection (Charting)

Two reviewers used the search strategy, and independently searched pre-identified databases. We first screened out irrelevant studies which mostly focused on stigma against HIV/AIDS, cognitive behavioral therapies, substance abuse/smoking, resilience scales, and clinical treatment related studies by reviewing titles and abstracts. We then imported the remaining studies, into RefWorks 2.0 database management software (2001) [27]. Duplicates were removed. We then screened titles and abstracts again and briefly scanned the full text to exclude studies not evaluating target outcomes. All studies that passed this exclusion process were included in the third stage of review for relevance by scanning title, abstracts and the full text for relevancy. At the next stage, we reviewed full-text articles for all the final included studies. Additionally, we added original studies which were referenced in included studies that cited their psychometric properties. We also checked the reference list of included studies for additional studies.

**Fig. 1:**
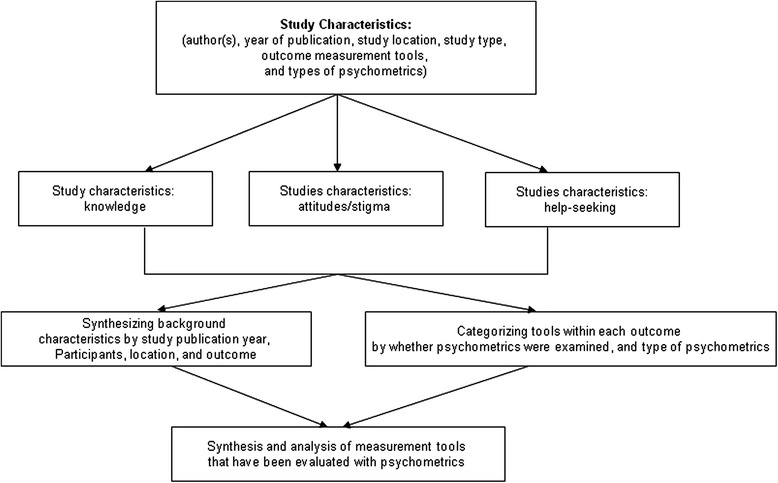
Charting process (data extraction process)

We applied “charting” techniques to conduct data extraction. For scoping reviews, “charting” is a data extraction technique to synthesize and interpret data by “sifting, charting and sorting materials according to key issues and themes” (page 26) [26, 28]. The key themes we followed in the “charting” of our data are the three outcome measures: knowledge, attitudes, and help-seeking, which was the base of the data categorization. We also charted data by year of publication, study location, study type, outcome measures, and types of psychometrics examined (e.g., reliability, validity, and responsiveness/sensitivity to change). The detailed charting process for this review is depicted in Fig. 1.

A data extraction form, developed in advance, was used for data extraction. We categorized studies into four types based on the extent of how psychometric properties were investigated and reported in the study: validation studies with evaluating psychometrics (any type) and/or responsiveness/sensitivity to change as the major purpose of the study (coded as P); studies evaluating effectiveness of interventions or survey studies evaluating psychometrics (any type) and/or responsiveness/sensitivity to change of the outcome measures (coded as I/P or S/P); studies just reporting but not evaluating psychometrics and/or responsiveness/sensitivity to change of the applied tool (coded as I/? or S/?); and studies mentioning the measurement tool applied but not reporting psychometrics (coded as I or S), including studies that quoted psychometrics from other studies, but did not evaluate it in the current study. We then sorted and defined the data by measures on knowledge, attitudes/stigma towards mental illness, and help seeking respectively, listed authors who first applied the tool, and calculated the number of psychometrics studies for each outcome measurement. In addition, we collated all psychometrics studies in separate tables. Figure 1 illustrates this process.

Once this charting process was completed, we reviewed all included studies, developed and populated tables, and created charts and figures according to the above described typology in an Excel spreadsheet. To help ensure consistency in interpretation and validity of the final results, one of the reviewers read and charted all included studies. Then the second reviewer checked all tables and compared and discussed the results with the first reviewer and they came to a consensus on the interpretation of the results. One methodology expert and two content experts were invited to help make the final decision when consensus was not reached between the two reviewers.

## Results

**Fig. 2:**
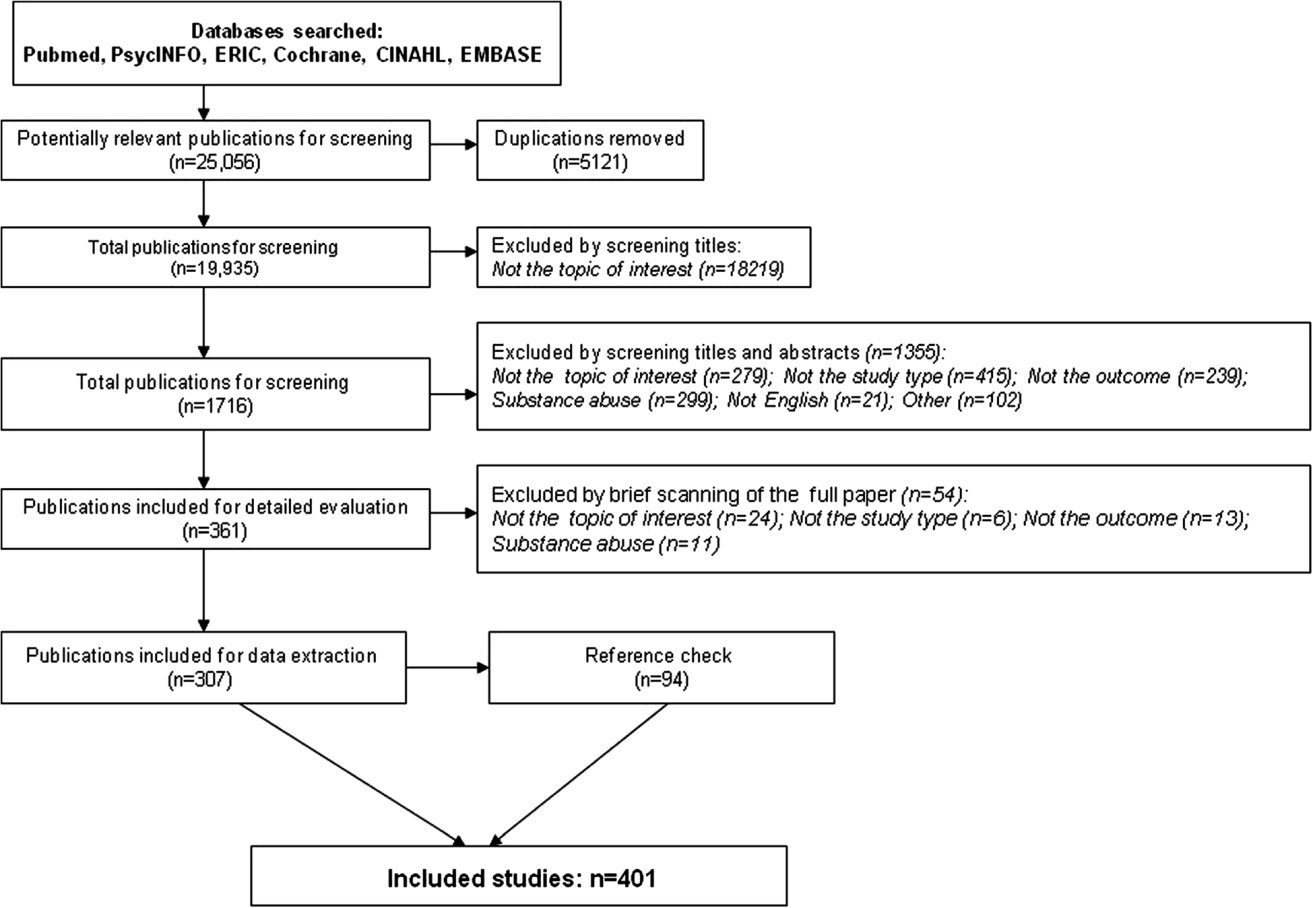
Search results

Figure 2 presents the flow chart of the screening process and final included studies. A total of 401 studies^1^ were identified that met study criteria, including 113 studies containing 69 knowledge measures, 307 studies containing 111 stigma measures, and 91 studies containing 35 help-seeking measures. Measures that modified and applied the concepts of the original ones were not counted as a new measure in our review. Out of the 401 studies, 130 validation studies reported and evaluated psychometrics (reliability, validity and/or the responsiveness/sensitivity to change) of the measures applied (P, I/P, or S/P), including14 knowledge studies (14 measures) (Table 1) [29–42], 102 stigma/attitudes studies (65 measures) (Table 2) [35, 36, 39, 43– 142], and 19 help-seeking studies (10 measures) (Table 3) [35, 101, 143–159]. These 3 tables summarized characteristics of validated studies, however we only listed authors who developed or first applied the measures although we included and summarized study results from other authors. Of these 130 studies, 5 studies also evaluated and reported responsiveness/sensitivity to change. Total number of studies for each sub-category may not necessarily match the total number of included studies because some studies tested more than one measurement tool in one study.

**Table 1:** Psychometrics of knowledge measures

Measures	Developer/Author	Reliability	Validity & responsiveness	Content
1.Knowledge about Schizophrenia Questionnaire (KASQ)	Ascher-Svanum, 1999 [29]	KR-20^a^ = .85, .89	R; CT	S
		r it^a^ = .46; .51		
		r^b^ = .83 (*p* < .005)		
2. Knowledge About Schizophrenia Test (KAST)	Compton et al., 2007 [30]	KR-20^a^ = .82 (.45-.78)	CS; CT; CR	S
3. Multiple-Choice Knowledge of Mental Illnesses Test (MC-KOMIT)	Compton et al., 2011 [31]	α^a^ = .68-.75	R; CS; CT	G
		r^b^ = .79 (*p* < .001)		
4. Mental Health Knowledge Schedule (MAKS);	Evans-Lacko et al., 2010 [32]	α^a^ = .65	CT	G
		Lin’s Pc ^b^ = .71;		
		k^b^ = .57-.87		
5. Depression Multiple Choice Question (MCQ)	Gabriel & Violato, 2009 [33]	α^a^ = .68	CT; CV; FA	D
6. Depression literacy scale (D-Lit)	Kiropoulos et al., 2011 [34]	α^a^ = ..88, .92		D
		r^b^ = .78, .80 (*p* < 0.001)		
	Gulliver et al., 2012 [35]	α^a^ = .70		
		r^b^ = .71 (*p* = .02)		
7. Anxiety Literacy Questionnaire (A-Lit)	Gulliver et al., 2012 [35]	α^a^ = .76;		A
		r^b^ = .83 (*p* = .003)		
8. Test of Knowledge About ADHD (KADD)	Hepperlen et al., 2002 [36]	α^a^ = .81-.82;	FA; CT	ADHD
9. Knowledge about Depression and Mania Inventory (KDMI)	Kronmuller et al., 2008 [37]	α^a^ = .89 (.76-.81);	CC/CR; CT; D; R	D
		r it^a^ = .36-.43		
10. Journey of Hope (JOH) Outcome Survey	Pickett-Schenk et al., 2000 [38]	α^a^ = .75-.83	CS; FA	G
11. Knowledge of Mental Disorders (KMD)	Serra et al., 2013 [39]	α^a^ = .588	CS; FA	G
12. Adolescent Depression Knowledge Questionnaire (ADKQ)	Shelley et al., 2014 [40]	α^a^ = .89	FA	D
13. Mental health disorder recognition questionnaire (MDRQ)	Swami et al., 2011 [41]	k^c^ = .94, .96	CS; CV	G
14. Mental Health Knowledge Questionnaire (MHKQ)	Wang et al., 2013 [42]	α^a^ = .69	FA	G

^a^internal consistency reliability: Cronbach alpha (α), Kuder-Richardson 20 (KR-20), item to total correlation (r it) ; ^b^test-retest reliability: weighted kappa (k), Pearson correlation coefficient (r), Lin’s Pc; ^c^ inter-rater reliability: weighted kappa (k)

*CT* Content validity, *CS* Construct validity, *CR* Criterion validity, *CV* Convergent validity, *CC* Concurrent validity, *D* Discriminant validity, *FA* Factor analysis, *R* Responsiveness

*G* general knowledge, *D* Depression, *S* Schizophrenia, *ADHD* Attention Deficit Hyperactivity Disorder, *A* Anxiety

**Table 2:** Psychometrics of stigma/attitudes measures

Measures	Developer/Author	Reliability	Validity & responsiveness	Content
1. Social Distance (SD)	Bogardus, 1925 [49]			
Link Social Distance scale (1987)	Link et al., 1987 [107]	α^a^ = .74; .75; .92	CS; CR/CV; D; FA	A
Bogardus Social Distance Scale (modified)	Angermeyer & Matschinger ., 2003 [44]	α^a^ = .90	FA	
Social distance scale	Link, 1983 [104]	α^a^ = .85, .91;	CS	
Reported and Intended Behaviour Scale (RIBS)	Evans-Lacko & Rose et al., 2011 [69]	α^a^ = .85;		
		k^b^ = .75		
RIBS- Japanese (RIBS-J)	Yamaguchi et al., 2014 [141]	α^a^ = .83	CC; FA	
		Lin’s Pc^b^ = .71		
Social Contact Scale	Jackson & Heatherington, 2006 [91]	α = .55-.75	FA	
The Social Supports Acceptance Scale (SSAS)	Mansouri & Dowell, 1989 [114]	α = .80-.94	CS; R	
2. Opinions about Mental Illness (OMI)	Cohen & Struening, 1962 [59]		CS; FA	A
OMI	Struening & Cohen, 1963 [128]	α^a^ = .299-.80	FA	
OMI in Chinese Community scale (OMICC)	Ng & Chan, 2000 [123]	α^a^ = .87 (.43-.72)	FA	
3. Community Attitudes towards Mental Illness (CAMI)	Taylor & Dear, 1981 [134]	α^a^ = .62-.90	CS; D; FA	A
Fear and Behavioural Intentions (FABI)	Svensson et al., 2011 [131]	α^a^ = .80		
		k^b^ = .29-.54		
Mental Health Attitude Survey for Police	Clayfield et al., 2011 [58]	α^a^ = .87	CS; CV; FA	
4. Devaluation-Discrimination tool (DD)	Link 1987 [105]	α^a^ = .73-.83	CS	A
Perceived Discrimination Devaluation (PDD)	Interian et al., 2010 [89]	α^a^ = .80	CS; CV/CR; FA	
Public stigma	Moses, 2009 [121]	α^a^ = .76	CV; CS; D	
		K^c^ = .79-.90		
Stigma-Devaluation Scale (SDS)	Dalky, 2012 [64]	α^a^ = .87	FA	
Depression is a Matter of Will	Aromaa et al., 2010 [45]		CS; FA	
5. Depression Stigma scale (DSS)	Griffiths et al., 2004 [79]	α^a^ = .75-.82	DS; CV; D; FA	A
		r^b^ = .86 (*p* = .001)		
6. Attribution Questionnaire (AQ)	Corrigan et al., 2003 [61]	α^a^ = .70-.96	CS; FA	A
AQ-27	Brown, 2008 [53]	α^a^ = .60-.93	CV; FA	
		ICC^b^ = .72-.90		
r-AQ	Pinto et al., 2012 [125]	α^a^ = .70	FA	
7. Internalized Stigma of Mental Illness (ISMI)	Ritsher et al., 2003 [126]	α^a^ = .84-.98	CS; CC; D; FA; P	C
		r^b^ = .92 (.61-.91) (*p* < .05) ICC^b^ = .78		
Parents’ Internalized Stigma of Mental Illness Scale (PISMI)	Zisman-llani et al., 2013 [142]	α^a^ = .61-.78	FA	
ISMI Chinese (ISMIS-C)	Lien et al., 2014 [103]	α^a^ = .90;	CS; FA	
		ICC = .36-.73		
ISMI-10	Boyd et al., 2014 [24]	α^a^ = .75	CT; CC; CS	
8. Perceived dangerousness (PD)	Link, et al., 1987 [106]	α^a^ = .85	CS	A
Link Stigma Scale (dangerousness)	Bagley & King, 2005 [46]	α^a^ > .80	CS; CR; D	
Dangerousness Scale (DS)	Penn et al., 1994 [124]	α^a^ = .78	CS	
9. British Omnibus National Survey (ONS)	Kobau et al., 2010 [100]	α^a^ = .66-.69	CV; CC; FA	A
Changing Mind	Svensson et al., 2011 [131]	α^a^ = .19-.46		
		r^b^:Poor to moderate		
10. Self-stigma of Seeking Help (SSOSH)	Vogel et al., 2006 [136]	α^a^ = .88	CS; P; D; FA	C
		r^b^ = .72		
11. Self-stigma of Mental Illness (SSMIS)	Corrigan et al., 2006 [63]	α^a^ = .64-.91;	CS; D	C
		r^b^ = .62-.82		
SSMIS-Short Form	Corrigan et al., 2012 [62]	α^a^ = .22-.87	CS; D	
12. Attitudes to Mental Illness Questionnaire (AMID)	Luty et al., 2006 [108]	r^b^ = .70; .93	CC; FA	A
13. Stigma Scale for Receiving Psychological Help (SSRPH)	Komiya et al., 2000 [101]	α^a^ = .72	CS; CR; FA	D
14. Affective Reaction Scale	Penn et al., 1994 [124]	α^a^ = .86	CS	A
15. Discrimination and Stigma Scale (DISC)	Brohan et al., 2013 [52]	α^a^ = .78;	CV; DV	B
		Lin’s Pc ^b^ = .88, 89 (*p* < .001)		
		k^b^ = 0.45-0.89		
		K^c^ = .62-.97		
Questionnaire on Anticipated Discrimination (QUAD)	Gabbidon et al., 2013 [72]	α^a^ = .86;	CV	B
		Lin’s Pc^c^ = .81		
		k^b^ = .41-.80		
16. Mental Illness: Clinician’s Attitudes (MICA)	Kassam et al., 2010 [94]	α^a^ = .79	CV; DV; FA; R	A
		Lin’s Pc ^b^ = .80 (*p* < .001)		
MICA-v4	Gabbidon et al., 2013 [73]	α^a^ = .72;	CV; FA	
		r it ^a^ ≥ .2		
17. Day’s Mental Illness Scale (DMISS)	Day et al., 2007 [65]	α^a^ = .71-.86	CS; FA	A
18. ADHD Stigma Questionnaire (ASQ)	Kellison et al., 2010 [98]	α^a^ = .55-.93;	CS; CV; DV; FA	A
		ICC^b^ = .71(.55-.73)		
Stigmatization towards Adults ADHD	Fuermaier et al., 2012 [71]	α^a^ = .91 (.61-.87)	CS	
19. Rejection Experiences	Link, 1987 [105]	α^a^ = .73-.85	CS; CR; CV; D	B
		K^c^ = .79-.90		
20. Generalized Anxiety Stigma Scale (GASS)	Griffiths et al., 2011 [81]	α^a^ = .86-0.91	CR; CS; CV; D; FA	A
		r^b^ = .55,.58,.91 (*p* < .0001, .001)		
21. Relatives’ opinions toward Schizophrenia	Magliano et al., 1999 [111]	α^a^ = .56-.66;	CS; FA	A
		K^b^ = .36-.84		
Questionnaire on the Opinions About Mental Illness (QO)	Magliano et al., 2004 [112]	α^a^ = .42-.72;	FA	A
		k^c^ = .50-1.0		
22. EMIC	Chowdhury et al., 2000 [57]	α^a^ = .66-.76;		A
		K^c^ = .77-.89		
23. Stigma Concerns about Mental Health Care (SCAMHC)	Interian et al., 2010 [89]	α^a^ = .69	CS; CV; CR; FA	D
24. Latino Scale for Antidepressant Scale (LSAS)	Interian et al., 2010 [89]	α^a^ = .66	CS; CV; CT; FA	D
25. Devaluation of Consumer Families Scale	Struening et al., 2001 [129]	α^a^ = .82	CV; FA	A
26. Devaluation of consumers scale	Struening et al., 2001 [129]	α^a^ = .71-.77	CV; FA	A
27. Consumer Experiences of Stigma Questionnaire (CESQ)	Bagley & King, 2005 [46]	α^a^ = .79-.82	CC; CS; CR; D; FA	B
28. Attitudes towards Depression and Its Treatment (ATDT)	Gabriel & Violato, 2010 [74]	α^a^ = .57-.79	CT; FA	A
29. Stigmatization Scale	Harvey, 2001 [83]	α^a^ = .90, .94	CS; D; FA; CR	B
30. Psychiatric Skepticism Scale (PSS)	Swami & Furnham, 2011 [132]	α^a^ = .92;.94	CS; FA	D
31. Emotional Reactions	Angermeyer & Matschinger, 2003 [44]		CS; FA	A
32. Labeling of mental illness	Angermeyer & Matschinger, 2003 [44]	k^c^ = 0.85		A
33. Personal Attributes	Angermeyer & Matschinger, 2003 [44]		CS; FA	A
34. Depression Attitude Questionnaire (DAQ)	Botega et al., 1992 [50]		FA	A
R-DAQ	Haddad et al., 2015 [82]	α^a^ = .84;	CT; CS; CV; FA	A
		ICC^b^ = .62		
35. Attitudes Toward psychiatry-30	Burra et al., 1982 [55]	r it ^a^ = .10-.64;	CC	D
		Split-half r^a^ = .89, .90;		
		ICC^b^ = .51-.87		
36. Opening Minds Scale for Health Care Providers (OMS-HC)	Kassam et al., 2012 [97]	α^a^ = .78; .79; .82	CS; FA; CT; R	A
		r it ^a^ = -.13-.57;		
		ICC^b^ = .66 (*p* < .001)		
37. Stigma Scale	King et al., 2007 [99]	α^a^ = .87 (.64-.87);	CS; CC; FA	A
		k^b^ = .41-.71		
Chinese Stigma Scale (CSS)	Ho et al., 2015 [87]	α^a^ = .83 (.58-.84)	CC; FA	A
38. Stigma Experiences Scale	Stuart et al., 2005 [130]	α^a^ = .91	CS	B
		KR-20^a^ = .83;		
39. Attitudes Toward Serious Mental Illness Scale-Adolescent	Watson et al., 2005 [139]		FA	A
40. Self reported prejudiced attitudes	Andersson et al., 2010 [43]	α^a^ = .78	FA	A
41. Self-Stigma of Depression Scale	Barney et al., 2010 [47]	α^a^ = .87	CS; CV; FA	C
		ICC^b^ = .63 (*p* = .000)		
42. Employer Attitude Questionnaire (EAQ)	Diksa & Rogers, 1996 [66]		FA	A
43. 15-Item Stigma Questionnaire	Gibbons et al., 2012 [75]	α^a^ = .85;	CC; CS; CV; CT	B
		ICC^b^ = .75		
44. Attitudes of Nursing Staff towards Co-Workers Returning from Psychiatric and Physical Illnesses	Glozier et al., 2006 [76]	α^a^ = .76-.88	CS	A
45. Self-Esteem and Stigma Questionnaire (SESQ)	Hayward et al., 2002 [84]	α^a^ = .71-.79;	CS	A
		r^b^ = .63 (*p* < .0001)		
46. Test of Knowledge About ADHD (KADD)	Hepperlen et al., 2002 [36]	α^a^ = .81-.82;	FA	A
47. Beliefs toward Mental Illness (BMI)	Hirai & Clum, 2000 [86]	α^a^ = .91;	CS; CC; FA	A
		r it = (.22 < r < .72)		
48. Depression Self-Stigma Scale (DSSS)	Kanter, 2008 [92]	α^a^ = .79-.95;	CS; CC; FA	C
		r it = .44-.83		
49. General Attitude Questionnaire	Lam et al., 2005 [102]	α^a^ = .88-.93		A
		r^b^ = .72-.94		
50. Secrecy	Link, 1987 [105]	α^a^ = .73-.83	CS	C
51. Withdrawal	Link, 1987 [105]	α = .73-.83	CS	C
52. Attitudes to Severe Mental Illness (ASMI)	Madianos et al., 2012 [110]	α^a^ = .88(.79-.86);	CS; P; FA	A
		r^b^ = .89-.92 (*p* < .0001)		
53. Affiliate Self-Stigma Scale	Mak & Cheung et al., 2008 [113]	α^a^ = .94-.95;	CS; P; FA	C
		r it = .51-.81		
Self-Stigma Scale-Short (SSS-S)	Wu et al., 2015 [140]	α^a^ = .95	CC; CS; FA	C
54. Knowledge Test of Mental Illness (KT)	Michaels & Corrigan, 2013 [116]	r^b^ = .50-.70 (*p* < .05; .001)	CC; CS	A
55. Attitudes Toward Social Competence and Integration of People with Mental Illness	Minnebo & Acker et al., 2004 [117]	α^a^ = .77; .79	FA	A
56. Client Attitude Questionnaire	Morrison & Becker, 1975 [120]	r^b^ = .90; .93		?
57. Libertarian Mental Health Ideology Scale (LMHIS)	Nevid & Morrison, 1980 [122]	α^a^ = .81-.94	CS; FA	D
58. Personal stigma scale	Schneider et al., 2011 [127]	α^a^ = .62-.92	FA	A
59. Child stigma scale	Moses, 2009 [121]	α^a^ = .81	CV; CS; D	C
		k^c^ = .79-.90		
60. Beliefs and attitudes toward people diagnosed with psychosis	Serra et al., 2013 [39]	α^a^ = .69	FA	A
61. Stigma of Depression Scale	Vega et al., 2010 [135]	α^a^ = .69	FA	A
62. Perceptions of Stigmatization by Others for Seeking Help (PROSH)	Vogel et al., 2009 [137]	α^a^ = .78-.91	CS; CC; FA	D
		r^b^ = .82 (*p* < .001)		
63. The Stigma Inventory for Mental Illness	Karidi et al., 2014 [93]	α^a^ = .90 (.75, .85);	CT; CS; CC; FA	C
		r^b^ = .80 (*p* < .001)		
64. Peer Mental Health Stigmatization Scale	McKeague et al., 2015 [115]	α^a^ = .80 (.70, .75);	CT; CS; D; FA	A
		r^b^ = .65, 75		
65. Endorsed and Anticipated Stigma Inventory (EASI)	Vogt et al., 2014 [138]	r it = .47-.75	CT; CV; D; FA	A & D

^a^Internal consistency reliability: Cronbach alpha (α), Kuder-Richardson 20 (KR-20), item to total correlation (r it), split-half reliability; ^b^test-retest reliability: intraclass correlation coefficient (ICC), weighted kappa (k), Pearson correlation coefficient (r), Lin’s Pc; ^c^ inter-rater reliability: weighted kappa (k), Lin’s Pc

*CT* Content validity, *CS* Construct validity, *CR* Criterion validity, *CV* Convergent validity, *CC* Concurrent validity, *D* Discriminant validity, *FA* Factor analysis, *R* Responsiveness, *DV* Divergent validity, *P* Predictive validity

*A* Stigma against mental illness or the mentally ill, *B* Experienced stigma, *C* self-stigma, *D* stigma against help-seeking, treatment; mental health institution or psychiatry, ? not reported

**Table 3:** Psychometrics of help-seeking measures

Measures	Author/developer	Reliability	Validity	Content
1. Attitudes towards help-seeking scale (with various modified versions)	Fischer & Turner, 1970 [143]	α^a^ = .83; .86	FA; CS	A/H
		r it = -.58 - .56 (*p* < .0001)		
		r^b^ = .73-.89		
Attitudes Toward Seeking Professional Psychological Help Scale (ATSPPH)	Fischer & Farina, 1995 [147]	α^a^ = .77-.98;.84;.90	FA; CS; CR	
		r it ^a^ = .54;		
		r^b^ = .80		
ATSPPH-SF	Elhai, et al., 2008 [146]	α^a^ = .69; .77-.78; .84	FA; CS	
		r it ^a^ > .40		
		r^b^ = .64 (*p* = .045)		
2. Intention of Seeking Counseling Inventory (ISCI)	Cepeda-Benito & Short, 1998 [144]	α^a^ = .89	FA; CS	I
3. General Help Seeking Questionnaire (GHSQ)	Deane et al., 2001 [145]	α^a^ = .67, .76, .82	FA	I
	Gulliver et al., 2012 [35]	α^a^ = .57-.77;		
		r^b^ = .42-.91 (*p* < 0.001)		
	Wilson et al., 2005 [159]	α^a^ = .70-.85;	P; CV; DV	
		ICC^b^ = .86-.92		
4. Jorm Mental health literacy survey (items on attitudes/beliefs towards treatment)	Jorm, Blewitt et al., 2005 [149]	K^c^ = 0.15-1.00		A/T
	Jorm, Mackinnon et al., 2005 [150]		FA	
	Reavley et al., 2014 [154]		CS	
5. Help Seeking Intentions	Lee et al., 2014 [151]	α^a^ = .74, .76	FA	I
6. The New Inventory of Attitudes Towards Seeking Mental Health Services (IASMHS)	Mackenzie et al., 2004 [152]	α^a^ = .87 (.76-.82)	FA; CS	A/H
		r^b^ = .64-.91 (*p* < 0.01)		
7. Help-Seeking Attitude Scale (HSAS)	Nickerson et al., 1994 [153]	α^a^ = .87	CC	A/H
8. Scale of Attitudes Toward Seeking Psychological Help for Secondary Students (ASPH-S)	Sahin & Uyar, 2011 [156]	α^a^ = .85 (.59-.81)	FA	A/H
		r it ^a^ = .41-.57		
		ICC^b^ = .81		
9. Help Seeking Acceptability (HSA)	Schmeelk-Cone et al., 2012 [157]	α^a^ = .84-.88	FA; CS	A/H
		r it ^a^ = .81-.85		
10. Parental Attitudes Toward Psychological Services Inventory (PATPSI) (based on ATSPPH)	Turner, 2012 [158]	α^a^ = .72-.92	FA; CS	A/H
		ICC^b^ = .66-.90		

^a^Internal consistency reliability: Cronbach alpha (α), item to total correlation (r it); ^b^test-retest reliability: intraclass correlation coefficient (ICC), Pearson correlation coefficient (r); ^c^ inter-rater reliability: weighted kappa (k)

*CT* Content validity, *CS* Construct validity, *CR* Criterion validity, *CV* Convergent validity, *CC* Concurrent validity, *D* Discriminant validity, *FA* Factor analysis, *DV* Divergent validity, *P* Predictive validity

*A/H* Beliefs/Attitudes towards help-seeking, *I* help-seeking intentions, *A/T* beliefs/attitudes towards treatment

**Fig. 3:**
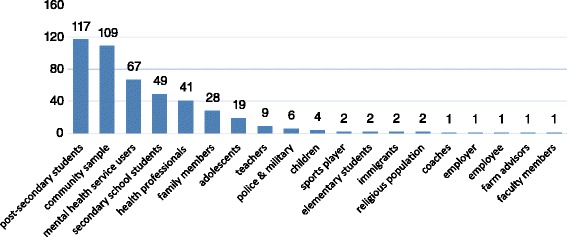
Study participants by study numbers

**Fig. 4:**
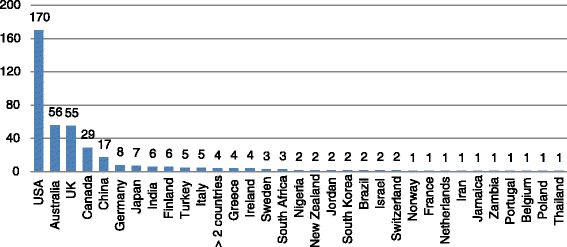
Study sites by study numbers

**Fig. 5:**
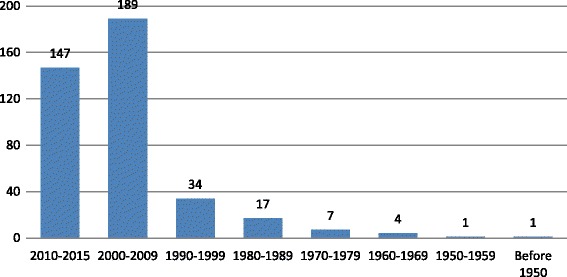
Publication dates by study numbers

**Fig. 6:**
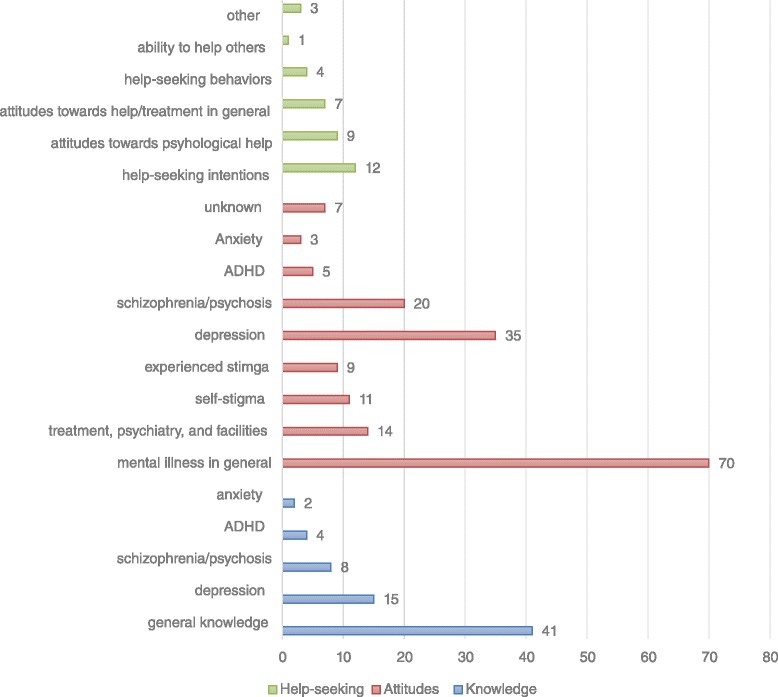
Measure content in each outcome (knowledge, attitudes and help-seeking) by study numbers

Study characteristics, such as study participants, locations, publication dates, and tool outcomes are reported in Figs. 3, 4, 5 and 6. Studies were conducted in 32 countries, with the United States of America most commonly, followed by Australia and Canada. Study participants were mainly post-secondary students, especially students in psychology or related professions, followed by the general public, and mental health service users (e.g., patients and their families). Most of the studies (n = 337) were published after the year 2000.

### Knowledge measures

The most widely used knowledge measures (by the number of studies in which the measure was applied) include the Mental Health Literacy Questionnaire (MHLQ) by Jorm and colleagues (1997) [160], Mental Health Knowledge Schedule (MAKS) [32], the World Psychiatric Association (WPA) “Open the Doors” (WPA-OD) questionnaire [161], Depression Literacy Scale (DLS) [79], Knowledge about Schizophrenia Questionnaire (KASQ) [29], Schizophrenia Knowledge Questionnaire (SKQ) [162], and In Our Voices (IOV) knowledge measure [163].

The 69 knowledge measures evaluated general knowledge about mental health, knowledge on specific disorders such as depression, schizophrenia/psychosis, ADHD, and anxiety disorders (Fig. 6). They used different approaches to measure knowledge. Some measures, such as those based on the approach by Jorm et al. (1997) [160] used the recognition of specific mental disorders (e.g., depression or anxiety) from the vignette description of symptoms. Other knowledge measures evaluated factual knowledge about mental illness with the true/false/don’t know approach. This includes fact-based tests on terminology, prevalence, causes, diagnosis, etiology, prognosis, consequences, and course of illness; and knowledge about recognition, support, employment, treatment/help-seeking/controllability, and recovery/coping, etc. (e.g. [29, 32, 33, 41, 79, 161–165]). One tool addressed the ability to distinguish mental illness from neurological or somatic illnesses (e.g. [39]). There were a number of measures combining stigma knowledge and mental health knowledge [95, 166–169]. Finally, some were self-evaluation measures of extent of knowledge (e.g. [170, 171]).

Of the 69 measures, psychometric properties were reported for 26 (38 %). And the rest of the 43 measures (62 %) had no psychometric properties reported. Of 26 measures with reported psychometrics, 14 measures were evaluated for psychometric properties, including 2 measures for responsiveness/sensitivity to change [29, 31]. These 14 measures evaluated general mental health knowledge (6 measures), depression (4 measures), schizophrenia (2 measures), ADHD (1 measure), and anxiety disorders (1 measure) (Table 1). The rest of the twelve measures only reported but didn’t evaluate psychometrics (internal consistency) and therefore we didn’t include them in Table 1.

Most knowledge measures applied self-report multiple choice answers (true, false, I don’t know/not sure), or vignettes with open-ended/closed questions (e.g. [172]), or used Likert-scale statements as self- evaluation formats.

### Stigma measures

Of all the stigma measures, the most widely used measures (by the number of studies where the measure was applied) include the Social Distance scale (SD) [49]; Opinions about Mental Health Illness (OMI) [59]; Community Attitudes towards Mental Illness (CAMI, a modified version of OMI) [134]; Devaluation-Discrimination (DD) [105]; Depression Stigma scale (DSS, also called Personal and Perceived Stigma of Mental Illness) [79]; Attribution Questionnaire (AQ) [61]; Internalized Stigma of Mental Illness (ISMI) [126]; and Perceived Dangerousness (PD) [106].

The 111 focus of the stigma/attitudes measures included: 1. stigma against mental illness or the mentally ill, such as social distance (the degree to which people are willing to accept the mentally ill in regular social life), personal stigma (participants’ personal attitudes toward people with mental illness) and perceived stigma (participants’ beliefs about others’ attitudes about mental illness); 2. self-stigma; 3. experienced stigma by mental health service users; 4. stigma against mental health treatment, psychiatry, help-seeking, or mental health care facilities. Further, some measures evaluated stigma against specific mental illnesses, such as depression, anxiety, ADHD, and schizophrenia/psychosis. Eleven studies (7 measures) did not report what aspects of stigma were measured (Fig. 6).

Social distance measures investigated issues such as a person’s willingness to engage the mentally ill in the workplace and the community (e.g., employment, renting, being neighbors, marriage) [46, 69, 106, 108, 124, 173]. Similarly, measures evaluating stigmatizing experiences by the mentally ill focused on challenges people with mental illness experience in family and social life [52, 75, 92, 99, 126, 130].

Measures evaluating personal and perceived stigma covered areas such as authoritarianism, benevolence, mental hygiene ideology, social restrictions to the mentally ill, and etiology [59, 134]. Other measures evaluated components such as stigma related to illness prevalence, consequences, dangerousness/threat, treatment and recovery of mental illness, or the social/family life, social responsibilities, human rights, intelligence [36, 74, 79, 81, 105, 111, 116, 135]. In addition, there were personal and perceived stigma measures focusing on emotional/rejection responses, willingness to help, and disclosure concerns [44, 48, 57, 61, 86, 98, 124].

Self-stigma measures mostly evaluated cognition such as self-esteem, self-confidence, self-satisfaction/concurrence, self-blame; negative emotions such as low pride of oneself, shame, embarrassment, sense of inadequacy, inferiority to others, helpless, pressure; and behaviors such as withdrawal, fear of seeking help, and secrecy [47, 63, 83, 92, 113, 121, 136].

Measures examining stigma against treatment/help-seeking/mental health care/medical model/psychiatry addressed perspectives and emotions. For example, some measures evaluated stigma towards help seeking (e.g., help-seeking as personal weakness; people seeking help being less likeable, disturbed, posed risks to others, and should hide the fact of seeking help) [101, 137]. Other tools [55, 122, 132] investigated stigma toward psychiatry, for example, skepticism towards psychiatry; and stereotypes of psychiatrists, psychiatric hospitals, patients, and psychiatric treatments. Some tools measured emotional responses (e.g., fear, discomfort and embarrassment) to psychological services and mental health care [89, 144].

Eighty one (73 %) articles on stigma tools reported on some psychometrics. Sixty five measures had evidence of reliability (e.g., Crobach’s α; item-total correlations; KR-20; test-retest reliability; inter-rater reliability), validity (e.g, construct; concurrent; discriminant; convergent; predicative), or responsiveness/sensitivity to change (Table 2). Sixteen measures demonstrated only internal consistency, but none included discussions on how this was measured. Of these 81 measures, 48 evaluated stigma against the mental illness/ the mentally ill in general; 11 were self-stigma measures; 6 evaluated personally experienced stigma; and 12 evaluated stigma against mental health treatment (psychological and pharmacological), psychiatry, help-seeking, or mental health care facilities. One tool did not specify what it measured.

### Help-seeking measures

Of the 35 help-seeking related measures, the most widely used are: Attitudes towards Help-Seeking Scale (later modified as Attitudes toward Seeking Professional Psychological Help Scale) (ATSPPH) [143, 147]; the mental health literacy questionnaire (MHLQ) that contains items on beliefs towards treatments [160]; General Help Seeking Questionnaire (GHSQ) [145]; and Intention of Seeking Counseling Inventory (ISCI) [174].

These help-seeking measures evaluating help-seeking intentions; beliefs or attitudes towards seeking psychological help for mental health problems or illness; beliefs towards mental health help or treatment in general; actual help-seeking behaviors; help-seeking efficacy (e.g. knowledge about where and how to find help, and who to find help from); self-reported ability to help others; or multiple components such as help-seeking intentions, help-seeking efficacy, and barriers for help-seeking (Fig. 6).

Unlike measures of stigma against help-seeking described above, measures evaluating attitudes towards psychological help-seeking mostly addressed: recognition of need for psychological help; interpersonal openness; confidence in and trustworthiness of mental health practitioners [143]. Measures evaluating beliefs toward treatment mostly evaluated the perceived helpfulness, effectiveness or safety of various interventions [150, 175], or the myths of treatment [176]. One measure [177] added social norm items on perceived attitudes of others (e.g., friends, employer) on depression intervention.

Measures evaluating help-seeking intentions examined willingness, or preferences to seek help from different sources (e.g., friends, families, professionals, religion, or spiritual healers [151, 172, 178-182]. One measure [172] further evaluated 3 extra dimensions of help-seeking intentions: talking to the listed sources; comfort level of talking to these resources; and helpfulness of these resources. Another tool measured intention levels for various emotional/behavioral challenges among college students [144]. Two measures didn’t specify how intentions were measured [161, 183].

Measures addressing help-seeking behaviors evaluated whether help-seeking was sought, and if so, what type of help was sought (formal vs. informal) for both stressful events and mental illness [178, 181, 184, 185].

Ten measures had some psychometric evaluation such as internal consistency, reliability, factor analysis, construct validity, and criterion validity [143, 145, 147, 149–154, 156–159]. Details of the psychometrics of these 10 measures are presented in Table 3. The 10 measures with psychometrics addressed attitudes or beliefs towards help-seeking or treatments, and intentions for help-seeking (Table 3). Two measures reported the internal consistency of the tool [172, 181], but did not discuss how this were measured, and therefore were not included in the table. No psychometric properties were reported on measures of help-seeking behaviors.

## Discussion

We identified a number of significant issues for consideration. These are: 1) representativeness of study samples; 2) geographic weighting: 3) adequacy of measurement of mental health literacy (knowledge, stigma, and help-seeking).

### Representative samples

Almost half of the studies (n = 185) were conducted among adolescents and young adults, particularly with post-secondary students (n = 117) (Fig. 3) mostly from health related professions, such as psychology, social work, and nursing. This raises concerns about the generalizability of findings as participants are not representative of the general population.

Even within the context of postsecondary education, much less attention (only 9 studies) has been paid to the mental health literacy of educators, who are important role models and youth influencers in addressing mental health literacy [186]. Further research into mental health literacy should take these important factors into account.

### Geographic weighting

Research on the measurement of mental health literacy started as early as in late 1950’S but did not bloom until after 2000 (n = 336; 84 %) (Fig. 5). Most studies (Fig. 4) took place in developed countries, especially the United States (n = 170; 42 %). Although there is ethnic diversity in the United States, the United States cannot be seen to represent other cultures. Moreover, different countries have different health systems and this may impact the implementation of mental health literacy approaches. For studies conducted in developing countries, authors either adapted existing measures, or used the conceptual framework from developed countries to create their measures, however, very few discussed the process of translation or the method of cultural adaptation. Therefore, the impact of important contextual factors, such as culture, ethnicity, geographic locations, education and health system, on mental health literacy and its measurement is currently unknown.

### Adequacy of measurement

Our analysis suggests that, out of three outcomes of mental health literacy (knowledge, attitudes and help-seeking), most measures evaluated stigma (n = 111), followed by measures that evaluated knowledge (n = 69), and a smaller number of help-seeking (n = 33). Only a relatively small number of measures were validated in any way. Secondly, widely used measures are often not validated. For example, the WPA mental health knowledge questionnaire was applied in 9 studies but no research has been identified to analyze its psychometric properties except for internal consistency.

Given the high proportions of un-validated measures being applied, it was difficult to determine the value of the study results and not possible to conduct cross-study comparisons of different interventions. There is a pressing need to validate these measures before their application.

With the measures that have been validated, there has been no research identified that appraised the quality of psychometric studies, and therefore, we were not able to recommend which measures are better than others. Further, given that the measures included in this review vary in their content, purposes and quality (measurement properties), more advanced research, such as systematic reviews is needed to locate evidence-based measures for use. Consensus-based Standards for the Selection of Health Measurement Instruments (COSMIN) [187] has been developed to serve this purpose and could be adapted for use in the comparative evaluation of mental health literacy measures.

Further, our review did not identify any measures addressing knowledge of positive/good mental health. Future measures should investigate knowledge on how to obtain and maintain good health as this now is recognized as an important component of mental health literacy.

### Knowledge measures

Our findings indicate that the diagnostic vignette approach is widely used as a measure of mental health knowledge. However, a recent study in which diagnostic vignettes were compared against non-diagnostic vignettes showed an inability of participants to discriminate across “normal” and “ill” categories [188]. Further study to establish the validity of the diagnostic vignette evaluation approach as a measure of mental health knowledge is needed.

The myths and facts approach to measure knowledge has covered a wide range of aspects of mental health. However, we are unable to determine if there are different and developmentally appropriate knowledge components addressed at different points of the life-span among the current available measures.

### Stigma measures

The plethora of stigma measures, developed from numerous different ideological models (e.g., labeling theory [189]; attribution framework model [61]; cognitive behavioral model [190]; and social stigma model [191], has made evaluation of their validity in addressing stigma/attitudes challenging. The challenge has been to both validate each of the specific models and to determine which model may provide a better explanatory prediction for stigma or attitudes in different groups of people.

Further, only a few measures have targeted people’s emotional responses (n = 8) towards mental illness. This is an important area because stigma is associated with self-experience of unpleasant feelings about mental illness and this may influence how people interact with those with mental illness [9]. Only very recently has research measured the stigma experience of people with mental illness (n = 28 studies). This may provide a more comprehensive picture of how society treats people with mental illness. This may help to provide more concrete and useful information on how stigma interventions should be developed and delivered at both individual and community level.

Despite the challenges discussed above, this review has mapped out how stigma measures were developed and what they intended to measure, and this information may provide researchers and practitioners some guidance on which path to take either in designing their measures, or applying/ adapting existing measures, or developing related interventions or programs in the future.

### Help-seeking measures

Help-seeking behaviors are challenging to measure as they are influenced by many factors, such as knowledge about the behaviors, attitudes and beliefs towards the behaviors, social norms, and intentions to perform the given behavior [192]. Most help-seeking measures in this review have focused on attitudes towards help-seeking/treatment (n = 20) and intentions to seek help (n = 11), and very few measures (n = 4) directly measured actual help-seeking behaviors. Further, all 4 help-seeking behavior measures had no psychometric validation.

As Ajzen and Fishbein [191] pointed out, behaviors also may be influenced by self-expressed behavioral control which requires a person to have the skills, capacities, resources, and other important capacities needed to perform the behavior. However, we have not identified any measures to address these factors except for one tool measuring help-seeking efficacy (e.g. knowledge about where and how to find help, and who to find help from) [178].

### Limitations

We did not conduct a systematic review of the literature on available mental health literacy measures and therefore we are unable to come to conclusions about the quality of the studies applying the measures. We excluded non-English studies (n = 21 at the title and abstract screening stage) and may have missed important measures in other languages. We did not check the grey literature that includes non peer-reviewed publication or documents/reports produced on all levels of governments and academics, and therefore may have missed some eligible studies. We may also have mistakenly excluded some measures at the first screening stage of reviewing titles and abstracts where measures were not mentioned.

Additionally, although we tried to categorize and interpret measures within the category we attributed them to, some measures may contain items relevant to other categories, however we were unable to distinguish them with available information we have.

## Conclusions

Our review provides a compendium of available mental health literacy measurement measures for researchers and practitioners who are interested in applying existing measures or developing new measures that of particular relevance to their work. Because of how we selected eligible studies, our review further automatically forms a comprehensive dataset of current mental health literacy interventions for stakeholders to consider for their use. This review also identifies the many gaps in the field, such as the unbalanced application of knowledge and help-seeking evaluation measures compared to the stigma/attitudes measures, the yet-to-be validated measures in each outcome category, and the lack of measures that measure all components of mental health literacy concurrently. This gap identification could potentially guide future research work in the field. Further, we have conducted a thorough summary and synthesis of the psychometrics properties of included measures, and clarified the need to further investigate the quality of the psychometrics studies. At this stage, most of the measures were created without consultation with the intended participants such as students, teachers, patients or health providers. Future work should focus on joint collaboration across disciplines, between investigators and stakeholders and across more varied demographic and geographic groups.

## Additional file

Additional file 1:
**Supplementary files contain an example of search strategies in PubMed, and supplementary references of studies that applied mental health literacy measures but did not provide related psychometrics information.** (ZIP 97 kb)

## Footnotes

1 References of studies that applied mental health literacy measures but did not evaluate the psychometrics of the applied measure were included in the supplementary document unless they were specifically cited in the manuscript.

## References

[1] Costello EJ, Mustillo S, Keeler G, Angold A, Levin BL, Petrila J, Hennessy K (2004). Prevalence of psychiatric disorders in childhood and adolescence. Mental health services: a public health perspective.

[2] Kessler RC, Berglund P, Demler O, Jin R, Merikangas KR (2005). Lifetime prevalence and age of-onset distributions of DSM-IV disorders in the national comorbidity survey replication. Arch Gen Psychiatry.

[3] World Health Organization. WHO methods and data sources for global burden of disease estimates 2000-2011. World Health Organization. 2013. http://www.who.int/healthinfo/statistics/GlobalDALYmethods_2000_2011.pdf?ua=1. Accessed 13 Aug 2015.

[4] Bhatia S (2007). Childhood and adolescent depression. Am Fam Physician.

[5] Kessler RC, Foster CL, Saunders WB, Stang PE (1995). Social consequences of psychiatric disorders I: educational attainment. Am J Psychiatry.

[6] Mezuk B, Eaton WW, Albrecht S, Golden SH (2008). Depression and type 2 diabetes over the lifespan. Diabetes Care.

[7] Rugulies R (2002). Depression as a predictor for coronary heart disease: a review and meta-analysis. Am J Prev Med.

[8] Leaf PJ, Alegria M, Cohen P, Goodman SH, Horwitz SM, Hoven CW (1996). Mental health service use in the community and schools: results from the four-community MECA study. J Am Acad Child Adolesc Psychiatry.

[9] Thornicroft G (2007). Most people with mental illness are not treated. Lancet.

[10] Waddell C, McEwan K, Shepherd CA, Offord DR, Hua JM (2005). A public health strategy to improve the mental health of Canadian children. Can J Psychiatry.

[11] Gulliver A, Griffiths KM, Christensen H (2010). Perceived barriers and facilitators to mental health help-seeking in young people: a systematic review. BMC Psychiatry.

[12] Kelly CM, Jorm AF, Wright A (2007). Improving mental health literacy as a strategy to facilitate early intervention for mental disorders. Med J Austria.

[13] Kutcher S, Bagnell A, Wei Y (2015). Mental health literacy in secondary schools: a Canadian approach. Child Adolesc Psychiatr Clin N Am.

[14] Reavley NJ, Jorm AF. National survey of mental health literacy and stigma. Canberra: Department of health and ageing. 2011. http://pmhg.unimelb.edu.au/research_settings/general_community/?a=636496. Accessed 14 Aug 2015.

[15] Rusch N, Evans-Lacko S, Henderson C, Flach C, Thornicroft G (2011). Public knowledge and attitudes as predictors of help seeking and disclosure in mental illness. Psychiatr Serv.

[16] Corrigan PW, Watson AC (2003). Factors that explain how policy makers distribute resources to mental health services. Psychiatr Serv.

[17] Henderson C, Evans-Lacko S, Thornicroft G (2013). Mental illness stigma, help seeking, and public health programs. Am J Public Health.

[18] Wei Y, Hayden JA, Kutcher S, Zygmunt A, McGrath P (2013). The effectiveness of school mental health literacy programs to address knowledge, attitudes and help seeking among youth. Early Intervent Psychiatry.

[19] World Health Organization. The solid facts: health literacy. World Health Organization. 2013. http://www.euro.who.int/__data/assets/pdf_file/0008/190655/e96854.pdf. Accessed 15 Aug 2015.

[20] Schachter HM, Girardi A, Ly M, Lacroix D, Lumb AB, van Berkom J (2008). Effects of school-based interventions on mental health stigmatization: a systematic review. Child Adolesc Psychiatry Ment Health.

[21] Angermeyer MC, Dietrich S (2006). Public beliefs about and attitudes towards people with mental illness: a review of population studies. Acta Psychiatr Scand.

[22] Mittal D, Sullivan G, Chekuri L, Allee E, Corrigan PW (2012). Empirical studies of self-stigma reduction strategies: a critical review of the literature. Psychiatr Serv.

[23] Link BG, Yang LH, Phelan JC, Collins PY (2004). Measuring mental illness stigma. Schizophr Bull.

[24] Boyd JE, Adler EP, Otilingam PG, Peters T (2014). Internalized Stigma of Mental Illness (ISMI) scale: a multinational review. Compr Psychiatry.

[25] O'Connor M, Casey L, Clough B (2014). Measuring mental health literacy--a review of scale-based measures. J Ment Health.

[26] Arksey H, O’Malley L (2005). Scoping studies: towards a methodological framework. Int J Soc Res Methodol.

[27] RefWorks-COS PL, ProQuest LLC (2001). RefWorks.

[28] Ritchie J, Spencer L. Qualitative data analysis for applied policy research. In: Bryman A, R.G. Burgess, RG, editors. Analysing qualitative data. London: Routledge; 1994.

[29] Ascher-Svanum H (1999). Development and validation of a measure of patients’ knowledge about schizophrenia. Psychiatr Serv.

[30] Compton MT, Quintero L, Esterberg ML (2007). Assessing knowledge of schizophrenia: development and psychometric properties of a brief, multiple-choice knowledge test for use across various samples. Psychiatry Res.

[31] Compton MT, Hankerson-Dyson D, Broussard B (2011). Development, item analysis, and initial reliability and validity of a multiple-choice knowledge of mental illnesses test for lay samples. Psychiatry Res.

[32] Evans-Lacko S, Little K, Meltzer H, Rose D, Rhydderch D, Henderson C (2010). Development and psychometric properties of the mental health knowledge schedule. Can J Psychiatry.

[33] Gabriel A, Violato C (2009). The development of a knowledge test of depression and its treatment for patients suffering from non-psychotic depression: a psychometric assessment. BMC Psychiatry.

[34] Kiropoulos L, Griffiths KM, Blashki G (2011). Effects of a multilingual information website intervention on the levels of depression literacy and depression-related stigma in Greek-born and Italian-born immigrants living in Australia: a randomized controlled trial. J Med Internet Res.

[35] Gulliver A, Griffiths KM, Christensen H, Mackinnon A, Calear AL, Parsons A (2012). Internet-based interventions to promote mental health help-seeking in elite athletes: an exploratory randomized controlled trial. J Med Internet Res.

[36] Hepperlen TM, Clay DL, Henly GA, Barké CR, Hehperlen MH, Clay DL (2002). Measuring teacher attitudes and expectations toward students with ADHD: development of the test of knowledge about ADHD (KADD). J Atten Disord.

[37] Kronmüller KT, Saha R, Kratz B, Karr M, Hunt A, Mundt C (2008). Reliability and validity of the knowledge about depression and mania inventory. Psychopathology.

[38] Pickett-Schenk SA, Cook JA, Laris A (2000). Journey of hope program outcomes. Community Ment Health J.

[39] Serra M, Lai A, Buizza C, Pioli R, Preti A, Masala C (2013). Beliefs and attitudes among Italian high school students toward people with severe mental disorders. J Nerv Ment Dis.

[40] Shelley RH, Kastelic EA, Wilcox HC, Beaudry MB, Musei RJ, Heley K (2014). Achieving depression literacy: the adolescent depression knowledge questionnaire (ADKQ). Sch Ment Health.

[41] Swami V, Persaud R, Furnham A (2011). The recognition of mental health disorders and its association with psychiatric skepticism, knowledge of psychiatry, and the big five personality factors: an investigation using the overclaiming technique. Soc Psychiatry Psychiatr Epedemiol.

[42] Wang J, He Y, Jiang Q, Cai J, Wang W, Zeng Q (2013). Mental health literacy among residents in Shanghai. Shanghai Arch Psychiatry.

[43] Andersson HW, Bjørngaard JH, Silje Lill Kaspersen SL, Wang CEA, Skre I, Dahl T (2010). The effects of individual factors and school environment on mental health and prejudiced attitudes among Norwegian adolescents. Soc Psychiat Epidemiol.

[44] Angermeyer MC, Matschinger H (2003). The stigma of mental illness: effects of labeling on public attitudes towards people with mental disorder. Acta Psychiatr Scand.

[45] Aromaa E, Tolvanen A, Tuulari J, Wahlbeck K (2010). Attitudes towards people with mental disorders: the psychometric characteristics of a Finnish questionnaire. Soc Psychiat Epidemiol.

[46] Bagley C, King M (2005). Exploration of three stigma scales in 83 users of mental health services: implications for campaigns to reduce stigma. J Ment Health.

[47] Barney LJ, Griffiths KM, Christensen H, Jorm AF (2010). The self-stigma of depression scale (SSDS): development and psychometric evaluation of a new instrument. Int J Methods Psychiatr Res.

[48] Bell L, Long S, Garvan C, Bussing R (2011). The impact of teacher credentials on ADHD stigma perceptions. Psychol Sch.

[49] Bogardus E (1925). Measuring social distance. J Appl Sociol.

[50] Botega N, Mann A, Blizard R, Wilinson G (1992). General practitioners and depression – first use of the depression attitude questionnaire. Int J Methods Psychiatr Res.

[51] Boyd JE, Otilingam PG (2014). Brief version of the internalized stigma of mental illness (ISMI) scale: psychometric properties and relationship to depression, self esteem, recovery orientation, empowerment, and perceived devaluation and discrimination. Psychiatr Rehabil J.

[52] Brohan E, Clement S, Rose D, Sartorius N, Slade M, Thornicroft G (2013). Development and psychometric evaluation of the discrimination and stigma scale (DISC). Psychiatr Res.

[53] Brown SA (2008). Factors and measurement of mental illness stigma: a psychometric examination of the attribution questionnaire. Psychiatr Rehabil J.

[54] Bjorkman T, Svensson B, Lundberg B (2007). Experiences of stigma among people with severe mental illness. Reliability, acceptability and construct validity of the Swedish versions of two stigma scales measuring devaluation/discrimination and rejection experiences. Nord J Psychiatry.

[55] Burra P, Kalin R, Leichner P, Waldron JJ, Handforth JR, Jarrett FJ (1982). The ATP 30 – a scale for measuring medical students’ attitudes of psychiatry. Med Educ.

[56] Chang C, Wu T, Chen C, Wang J, Lin C (2014). Psychometric evaluation of the internalized stigma of mental illness scale for patients with mental illnesses: measurement invariance across time. PLoS One.

[57] Chowdhury AN, Sanyal D, Dutta SK, Banerjee S, De R, Bhattacharya K (2000). Stigma and mental illness: pilot study of laypersons and health care providers with the EMIC in rural west Bengal. India. Int Med J..

[58] Clayfield JC, Fletcher KE, Grudzinskas AJ (2011). Development and validation of the mental health attitude survey for police. Community Ment Health J.

[59] Cohen J, Struening EL (1962). Opinions about mental illness in the personnel of two large mental hospitals. J Abnorm Soc Psychol.

[60] Corrigan PW, Rowan D, Qreen A, Lundin R, River P, Uphoff'-Wasowski K (2002). Challenging two mental illness stigmas: personal responsibility and dangerousness. Schizophr Bull.

[61] Corrigan P, Markowitz FE, Watson A, Rowan D, Kubiak MA (2003). An attribution model of public discrimination towards persons with mental illness. J Health Soc Behav.

[62] Corrigan PW, Michaels PJ, Vega E, Gause M, Watson AC, Rusch N (2012). Self-stigma of mental illness scale-short form: reliability and validity. Psychiatry Res.

[63] Corrigan PW, Watson AC, Barr L (2006). The self-stigma of mental illness: implications for self-esteem and self-efficacy. J Soc Clin Psychol.

[64] Dalky HF (2012). Arabic translation and cultural adaptation of the stigma-devaluation scale in Jordan. J Ment Health.

[65] Day EN, Edgren K, Eshleman A (2007). Measuring stigma toward mental illness: development and application of the mental illness stigma scale. J Appl Soc Psychol.

[66] Diksa E, Rogers ES (1996). Employer concerns about hiring persons with psychiatric disability: results of the employer attitude questionnaire. Rehabil Couns Bull.

[67] Evans-Lacko S, Henderson C, Thornicroft G (2013). Public knowledge, attitudes and behavior regarding people with mental illness in England 2009-2012. Br J Psychiatry.

[68] Evans-Lacko S, London J, Japhet S, Rusch N, Flach C, Corker E (2012). Mass social contact interventions and their effect on mental health related stigma and intended discrimination. BMC Public Health.

[69] Evans-Lacko S, Rose D, Little K, Flach C, Rhydderch D, Henderson C (2011). Development and psychometric properties of the reported and intended behavior scale (RIBS): a stigma-related behavior measure. Epidemiol Psychiatr Sci.

[70] Friedrich B, Evans-Lacko S, London J, Rhydderch D, Henderson C, Thornicroft G (2013). Anti-stigma training for medical students: the education not discrimination project. Br J Psychiatry.

[71] Fuermaier ABM, Tucha L, Koerts J, Mueller AK, Lange KW, Tucha O (2012). Measurement of stigmatization towards Adults with Attention Deficit Hyperactivity Disorder. PLoS One.

[72] Gabbidon J, Brohan E, Clement S, Henderson RC, Thornicroft G, MIRIAD Study Group. The development and validation of the Questionnaire on Anticipated Discrimination (QUAD). BMC Psychiatry. 2013; 13: 297. http://www.biomedcentral.com/1471-244X/13/297. Accessed 14 Aug 2015. 10.1186/1471-244X-13-297 PMC4226195 24199691

[73] Gabbidon J, Clement S, Nieuwenhuizen A, Kassam A, Brohan E, Norman I (2013). Mental Illness: Clinicians’ Attitudes (MICA) Scale—Psychometric properties of a version for health care students and professionals. Psychiatry Res.

[74] Gabriel A, Violato C (2010). The development and psychometric assessment of an instrument to measure attitudes towards depression and its treatments in patients suffering from non-psychotic depression. J Affect Disord.

[75] Gibbons C, Dubois S, Morris K, Parker B, Maxwell H, Bédard M (2012). The development of a questionnaire to explore stigma from the perspective of individuals with serious mental illness. Can J Commun Ment Health.

[76] Glozier N, Hough C, Henderson M, Holland-Elliott K (2006). Attitudes of nursing staff towards co-workers returning from psychiatric and physical illnesses. Int J Soc Psychiatry.

[77] Granello DH, Pauley PS, Carmichael A (1999). Relationship of the media to attitudes toward people with mental illness. J Humanistic Couns Educ Dev.

[78] Granello DH, Pauley PS (2000). Television viewing habits and their relationship to tolerance toward people with mental illness. J Ment Health Couns.

[79] Griffiths KM, Christensen H, Jorm AF, Evans K, Groves C (2004). Effect of web-based depression literacy and cognitive-behavioural therapy interventions on stigmatizing attitudes to depression: randomized controlled trial. Br J Psychiatry.

[80] Griffiths KM, Christensen H, Jorm AF (2008). Predicators of depression stigma. BMC Psychiatry.

[81] Griffiths, KM, Batterham PJ, Barney L, Parsons A. The generalized anxiety stigma scale (GASS): psychometric properties in a community sample. BMC Psychiatry. 2011; 11: 184. http://www.biomedcentral.com/1471-244X/11/184. Accessed 14 Aug 2015. 10.1186/1471-244X-11-184 PMC3248354 22108099

[82] Haddad M, Menchetti M, McKeown E, Tylee A, Mann A (2015). The development and psychometric properties of a measure of clinicians’ attitudes to depression: the revised Depression Attitude Questionnaire (R-DAQ). BMC Psychiatry.

[83] Harvey RD (2001). Individual differences in the phenomenological impact of social stigma. J Soc Psychol.

[84] Hayward P, Wong G, Bright JA, Lam D (2002). Stigma and self-esteem in manic depression: an exploratory study. J Affect Disord.

[85] Hinkelmean L, Granello DH (2003). Biological sex, adherence to traditional gender roles, and attitudes toward persons with mental illness: an exploratory investigation. J Ment Health Couns.

[86] Hirai M, Clum GA (2000). Development, reliability, and validity of the beliefs toward mental illness scale. J Psychopathol Behav Assess.

[87] Ho AHY, Potash JS, Fong TCT, Ho VFL, Chen EYH, Lau RHW (2015). Psychometric properties of a Chinese version of the stigma scale: examining the complex experience of stigma and its relationship with self-esteem and depression among people living with mental illness in Hong Kong. Compr Psychiatry.

[88] Högberg T, Magnusson A, Ewertzon M, Lützén K (2008). Attitudes towards mental illness in Sweden: adaptation and development of the Community Attitudes towards Mental Illness questionnaire. Int J Ment Health Nurs.

[89] Interian A, Ang A, Gara MA, Link B, Rodriguez MA, Vega WA (2010). Stigma and depression treatment utilization among Latinos: utility of four stigma measures. Psychiatr Serv.

[90] Isaac F, Greenwood KM, Benedetto M (2012). Evaluating the psychometric properties of the attitudes towards depression and its treatments scale in an Australian sample. Patient Prefer Adherence.

[91] Jackson D, Heatherington L (2006). Young Jamaicans’ attitudes toward mental illness: experimental and demographic factors associated with social distance and stigmatizing opinions. J Community Psychol.

[92] Kanter JW, Rusch LC, Brondino MJ (2008). Depression Self-Stigma: a new measure and preliminary findings. J Nerv Ment Dis.

[93] Karidi MV, Vasilopoulou D, Savvidou E, Vitoratou S, Rabavilas AD, Stefanis CN (2014). Aspects of perceived stigma: the stigma inventory for mental illness, its development, latent structure and psychometric properties. Compr Psychiatry.

[94] Kassam A, Glozier N, Leese M, Henderson C, Thornicroft G (2010). Development and responsiveness of a scale to measure clinicians’ attitudes to people with mental illness (medical student version). Acta Psychiatr Scand.

[95] Kassam A, Glozier N, Leese M, Loughran J, Thornicroft G. A controlled trial of mental illness related stigma training for medical students. BMC Med Educ. 2011; 11: 51. http://www.biomedcentral.com/1472-6920/11/51. Accessed 14 Aug 2015. 10.1186/1472-6920-11-51 PMC3161004 21801355

[96] Kira IA, Ramaswamy V, Lewandowski L, Mohanesh J, Abdul-Khalek H. Psychometric assessment of the Arabic version of the Internalized Stigma of Mental Illness (ISMI) measure in a refugee population. Transcult Psychiatry. 2015; 1-23. doi: 10.1177/1363461515569755. 10.1177/1363461515569755 25665586

[97] Kassam A, Papish A, Modgill G, Patten S. The development and psychometric properties of a new scale to measure mental illness related stigma by health care providers: the opening minds scale for Health Care Providers (OMS-HC). BMC Psychiatry. 2012; 12: 62. http://www.biomedcentral.com/1471-244X/12/62. Accessed 14 Aug 2015. 10.1186/1471-244X-12-62 PMC3681304 22694771

[98] Kellison I, Bussing R, Bell L, Garvan C (2010). Assessment of stigma associated with attention-deficit hyperactivity disorder: psychometric evaluation of the ADHD stigma questionnaire. Psychiatry Res.

[99] King M, Dinos S, Shaw J, Watson R, Stevens S, Passetti F (2007). (2007). The stigma scale: development of a standardized measure of the stigma of mental illness. Br J Psychiatry.

[100] Kobau R, DiIorio C, Chapman D, Delvecchi P (2010). SAMHSA/CDC Mental Illness Stigma Panel Members. Attitudes about mental illness and its treatment: validation of a generic scale for public health surveillance of mental illness associated stigma. Community Ment Health J.

[101] Komiya N, Good GE, Sherrod NB (2000). Emotional openness as a predictor of college students’ attitudes toward seeking psychological help. J Couns Psychol.

[102] Lam DCK, Salkovskis PM, Warwick HM (2005). C. An experimental investigation of the impact of biological versus psychological explanations of the cause of “mental illness”. J Ment Health.

[103] Lien Y, Kao Y, Liu Y, Chang H, Tzeng N, Lu C (2014). Internalized stigma and stigma resistance among patients with mental Illness in Han Chinese population. Psychiatr Q.

[104] Link BG (1983). Reconsidering the social rejection of ex-mental patients: levels of attitudinal response. Am J Community Psychol.

[105] Link BG (1987). Understanding labeling effects in the area of mental disorders: an assessment of the effects of expectations of rejection. Am Sociol Rev.

[106] Link BG, Cullen FT, Frank J, Wozniak JF (1987). The social rejection of former mental patients: understanding why labels matter. AJS.

[107] Luca Pingani L, Forghieri M, Ferrari S, Ben-Zeev D, Artoni P, Mazzi F (2012). Stigma and discrimination toward mental illness: translation and validation of the Italian version of the attribution questionnaire-27 (AQ-27-I). Soc Psychiatry Psychiatr Epidemiol.

[108] Luty J, Fekadu D, Umoh O, Gallagher J (2006). Validation of a short instrument to measure stigmatized attitudes towards mental illness. Psychiatr Bull.

[109] Luty J, Umoh O, Sessay M, Sarkhel A (2007). Effectiveness of Changing Minds campaign factsheets in reducing stigmatised attitudes towards mental illness. Psychiatr Bull.

[110] Madianos M, Economou M, Peppou LE, Kallergis G, Rogakou E, Alevizopoulos G (2012). Measuring public attitudes to severe mental illness in Greece: development of a new scale. Eur J Psychiat.

[111] Magliano L, Marasco C, Guarneri M, Malangone C, Lacrimini G, Zanus P (1999). A new questionnaire assessing the opinions of the relatives of patients with schizophrenia on the causes and social consequences of the disorder: reliability and validity. Eur Psychiatry.

[112] Magliano L, Fiorillo A, Rosa C, Malangone C, Maj M (2004). Beliefs about schizophrenia in Italy: a comparative nationwide survey of the general public, mental health professionals, and patients’ relatives. Can J Psychiatry.

[113] Mak WWS, Cheung RYM (2008). Affiliate stigma among caregivers of people with intellectual disability or mental illness. J Appl Res Intellect Disabil.

[114] Mansouri L, Dowell DA (1989). Perceptions of stigma among the long-term mentally ill. Psychosoc Rehabil J.

[115] McKeague L, Hennessy E, O’Driscoll C, Heary C. Peer Mental Health Stigmatization Scale: psychometric properties of a questionnaire for children and adolescents. Child Adolesc Ment Health. 2015; doi: 10.1111/camh.12088. 10.1111/camh.12088 32680400

[116] Michaels PJ, Corrigan PW (2013). Measuring mental illness stigma with diminished social desirability effects. J Ment Health.

[117] Minnebo J, Acker AV (2004). Does television influence adolescents’ perceptions of and attitudes toward people with mental illness?. J Community Psychol.

[118] Modgill G, Knaak S, Kassam A, Szeto A. Opening minds stigma scale for health care providers (OMS-HC): examination of psychometric properties and responsiveness. BMC Psychiatry. 2014; 14:120. doi:10.1186/1471-244X-14-120. 10.1186/1471-244X-14-120 PMC4024210 24758158

[119] Morris R, Scott PA, Cocoman A, Chambers M, Guise V, Va¨lima¨ki M (2011). Is the Community Attitudes towards the Mentally Ill scale valid for use in the investigation of European nurses’ attitudes towards the mentally ill? A confirmatory factor analytic approach. J Adv Nurs.

[120] Morrison JK, Becker BE (1975). Seminar-induced change in a community psychiatric team’s reported attitudes toward “mental illness”. J Community Psychol.

[121] Moses T (2009). Stigma and self-concept among adolescents receiving mental health treatment. Am J Orthopsychiatry.

[122] Nevid JS, Morrison J (1980). Attitudes toward mental illness: the construction of the libertarian mental health ideology scale. J Humanistic Psychol.

[123] Ng P, Chan K (2000). Sex differences in opinion towards mental illness of secondary school students in Hong Kong. Int J Soc Psychiatry.

[124] Penn DL, Guynan K, Daily T, Spaulding WD, Garbin CP, Sullivan M (1994). Dispelling the stigma of schizophrenia: what sort of information is best?. Schizophr Bull.

[125] Pinto MD, Hickman R, Logsdon MC, Burant C (2012). Psychometric evaluation of the revised attribution questionnaire (r-AQ) to measure mental illness stigma in adolescents. J Nurs Meas.

[126] Ritsher JB, Otilingama PG, Grajales M (2003). Internalized stigma of mental illness: psychometric properties of a new measure. Psychiatry Res.

[127] Schneider J, Beeley C, Repper J (2011). Campaign appears to influence subjective experience of stigma. J Ment Health.

[128] Struening EL, Cohen J (1963). Factorial invariance and other psychometric characteristics of five opinions about mental illness factors. Educ Psychol Meas.

[129] Struening EL, Perlick DA, Link BG, Hellman FH, Herman D, Sirey JA (2001). The extent to which caregivers believe most people devalue consumers and their families. Psychiatr Serv.

[130] Stuart H, Milev R, Koller M. The inventory of stigmatizing experiences: its development and reliability. World Psychiatry. 2005; Suppl 4: 35-9.

[131] Svensson B, Markström U, Bejerholm U, Björkman T, Brunt D, Eklund M, et al. Test - retest reliability of two instruments for measuring public attitudes towards persons with mental illness. BMC Psychiatry. 2011; 11:11. http://www.biomedcentral.com/1471-244X/11/11. Accessed 14 Aug 2015. 10.1186/1471-244X-11-11 PMC3025948 21235749

[132] Swami V, Furnham A (2011). Preliminary examination of the psychometric properties of the Psychiatric Scepticism Scale. Scand J Psychol.

[133] Świtaj P, Paweł Grygiel P, Jacek Wciórka J, Humenny G, Anczewska M (2013). The stigma of subscale of the Consumer Experiences of Stigma Questionnaire (CESQ): a psychometric evaluation in Polish psychiatric patients. Compr Psychiatry.

[134] Taylor SM, Dear MJ (1981). Scaling community attitudes toward the mentally ill. Schizophr Bull.

[135] Vega WA, Rodriguez MA, Ang A (2010). Addressing stigma of depression in Latino primary care patients. Gen Hosp Psychiatry.

[136] Vogel DL, Wade NG, Haake S (2006). Measuring the self-stigma associated with seeking psychological help. J Couns Psychol.

[137] Vogel DL, Wade NG, Ascheman PL (2009). Measuring perceptions of stigmatization by others for seeking psychological help: reliability and validity of a new stigma scale with college students. J Couns Psychol.

[138] Vogt D, Di Leone BAL, Wang JM, Sayer NA, Pineles SL (2014). Endorsed and anticipated stigma inventory (EASI): a tool for assessing beliefs about mental illness and mental health treatment among military personnel and veterans. Psychol Serv.

[139] Watson AC, Miller FE, Lyons JS (2005). Adolescent attitudes toward serious mental illness. J Nerv Ment Dis.

[140] Wu TH, Chang CC, Chen CY, Wang JD, Lin CY (2015). Further psychometric evaluation of the self-stigma scale – short: measurement invariance across mental illness and gender. PLoS One.

[141] Yamaguchi S, Koike S, Watanabe K, Ando S (2014). Development of a Japanese version of the reported and intended behavior scale: reliability and validity. Psychiatry Clin Neurosci.

[142] Zisman-Ilani Y, Levy-Frank-Levy I, Hasson-Ohayon I, Kravetz S, Mashiach-Eizenberg M, Roe D (2013). Measuring the internalized stigma of parents of persons with a serious mental illness. Journal of Nervous and Mental Disease.

[143] Fischer EH, Turner JL (1970). Orientations to seeking professional help: development and research utility of an attitude scale. J Consult Clin Psychol.

[144] Cepeda-Benito A, Short P (1998). Self-concealment, avoidance of psychological services, and perceived likelihood of seeking professional help. J Couns Psychol.

[145] Deane FP, Wilson CJ, Ciarrochi J (2001). Suicidal ideation and help-negation: not just hopelessness or prior help. J Clin Psychol.

[146] Elhai JD, Schweinle W, Anderson SM (2008). Reliability and validity of the attitudes toward seeking professional psychological help scale – short form. Psychiatr Res.

[147] Fischer EH, Farina A (1995). Attitudes toward seeking professional psychological help: a shortened form and considerations for research. J Coll Stud Dev.

[148] Hatchett G (2006). Additional validation of the attitudes toward seeking professional psychological help scale. Psychol Rep.

[149] Jorm AF, Blewitt KA, Griffiths KM, Kitchener BA, Parslow RA (2005). Mental health first aid responses of the public: results from an Australian national survey. BMC Psychiatry.

[150] Jorm AF, Mackinnon A, Christensen H, Griffiths KM (2005). Structure of beliefs about the helpfulness of interventions for depression and schizophrenia. Soc Psychiatry Psychiatr Epidemiol.

[151] Lee J, Friesen BJ, Walker JS, Colman D, Donlan WE (2014). Youth’s help-seeking intentions for ADHD and depression: findings from a national survey. J Child Fam Stud.

[152] Mackenzie CS, Knox VJ, Gekoski WL, Macaulay HL (2004). An adaptation and extension of the attitudes toward seeking professional psychological help scale. J Appl Soc Psychol.

[153] Nickerson KJ, Helms JE, Terrell F (1994). Cultural mistrust, opinions about mental illness, and black students’ attitudes toward seeking psychological help from white counselors. J Couns Psychol.

[154] Reavley NJ, Morgan AJ, Jorm AF (2014). Development of scales to assess mental health literacy relating to recognition of and interventions for depression, anxiety disorders and schizophrenia/psychosis. Aust N Z J Psychiatry.

[155] Royal KD, Thompson JM (2012). Measuring protestant Christians’ willingness to seek professional psychological help for mental illness: a Rasch measurement analysis. J Psychol Christianity.

[156] Sahin H, Uyar SY. Turkish high school students’ attitudes towards seeking professional psychological help. US-China Educ Rev B 2. 2011; 251-9.

[157] Schmeelk-Cone K, Pisani AR, Petrova M, Wyman PA (2012). Three scales assessing high school students’ attitudes and perceived norms about seeking adult help for distress and suicide concerns. Suicide Life Threat Behav.

[158] Turner E (2012). The parental attitudes toward psychological services inventory: adaptation and development of an attitude scale. Community Ment Health J.

[159] Wilson CJ, Deane FP, Rickwood D (2005). Measuring help-seeking intentions: properties of the general help-seeking questionnaire. Can J Couns.

[160] Jorm AF, Korten AE, Jacomb PA, Christensen H, Rodgers B, Pollitt P (1997). “Mental health literacy”: a survey of the public’s ability to recognise mental disorders and their beliefs about the effectiveness of treatment. Med J Aust.

[161] Pinfold V, Toulmin H, Thornicroft G, Huxley P, Farmer P (2003). Reducing psychiatric stigma and discrimination: evaluation of educational interventions in UK secondary schools. Br J Psychiatry.

[162] Smith JV, Birchwood MJ (1987). Specific and non-specific effects of educational intervention with families living with a schizophrenic relative. Br J Psychiatry.

[163] Wood A, Wahl O (2006). Evaluating the effectiveness of a consumer-provided mental health recovery education presentation. Psychiatr Rehabil J.

[164] Stuart H, Arboleda-Florez J (2001). Community attitudes toward people with schizophrenia. Can J Psychiatry.

[165] Ghanizadeh A, Bahredar MJ, Moeini SR (2006). Knowledge and attitudes towards attention deficit hyperactivity disorder among elementary school teachers. Patient Educ Couns.

[166] DeSocio J, Stember L, Schrinsky J (2006). Teaching children about mental health and illness: a school nurse health education program. J Sch Nurs.

[167] Essler V, Arthur A, Stickley T (2006). Using a school-based intervention to challenge stigmatizing attitudes and promote mental health in teenagers. J Ment Health.

[168] Hoven C, Doan T, Musa GJ, Jaliashvili T, Duarte CS, Ovuga E (2008). Worldwide child and adolescent mental health begins with awareness: a preliminary assessment in nine countries. Int Rev Psychiatry.

[169] Ojanen M (1992). Attitudes towards Mental Patients. Int J Soc Psychiatry.

[170] Mullen A, Murray L, Happell B (2002). Multiple family group interventions in first episode psychosis: enhancing knowledge and understanding. Int J Ment Health Nurs.

[171] Williams B, Pow J (2007). Gender differences and mental health: an exploratory study of knowledge and attitudes to mental health among Scottish teenagers. Child Adolesc Ment Health.

[172] Cabassa LJ, Lester R, Zayas LH (2007). “It’s Like Being in a Labyrinth:” Hispanic immigrants’ perceptions of depression and attitudes toward treatments. J Immigr Minor Health.

[173] Link BG, Phelan JC, Bresnahan M, Stueve A, Pescosolido BA (1999). Public conceptions of mental illness: labels, causes, dangerousness, and social distance. Am J Public Health.

[174] Kelly AE, Achter JA (1995). Self-concealment and attitudes toward counseling in university students. J Couns Psychol.

[175] Hugo CJ, Boshoff DEL, Traut A, Zungu-Dirwayi N, Stein DJ (2003). Community attitudes toward and knowledge of mental illness in South Africa. Soc Psychiatry Psychiatr Epidemiol.

[176] Ozmen E, Ogel K, Aker T, Afsın Sagduyu A, Tamar D, Boratav C (2005). Public opinions and beliefs about the treatment of depression in urban Turkey. Soc Psychiatry Psychiatr Epidemiol.

[177] Iloabachie C, Wells C, Goodwina B, Baldwin M, Vanderplough-Booth K, Gladstone T (2011). Adolescent and parent experiences with a primary care/Internet-based depression prevention intervention (CATCH-IT). Gen Hosp Psychiatry.

[178] Costin DL, Mackinnon AJ, Griffiths KM, Batterham PJ, Bennett AJ, Bennett K (2009). Health e-Cards as a means of encouraging help seeking for depression among young adults: randomized controlled trial. J Med Internet Res.

[179] Nguyen QCX, Anderson LP (2005). Vietnamese Americans’ attitudes toward seeking mental health services: relation to cultural variables. J Community Psychol.

[180] Teng EJ, Friedman LC (2009). Increasing mental health awareness and appropriate service use in older Chinese Americans: a pilot intervention. Patient Educ Couns.

[181] Santor DA, Poulin C, LeBlanc JC, Kusumakar V (2007). Facilitating help seeking behavior and referrals for mental health difficulties in school aged boys and girls: a school-based intervention. J Youth Adolesc.

[182] Brown C, O’Conner K, Copeland VC, Grote N, Beach S, Battista D (2010). Depression stigma, race, and treatment seeking behavior and attitudes. J Community Psychol.

[183] Askell-Williams H, Lawson M, Murray-Harvey R (2007). Teaching and learning about mental illnesses. Int J Ment Health Promot.

[184] Chang H (2008). Help-seeking for stressful events among Chinese college students in Taiwan: roles of gender, prior history of counseling, and help-seeking attitudes. J Coll Stud Dev.

[185] Sussman LK, Robins LN, Earls F (1987). Treatment-seeking for depression by black and white Americans. Soc Sci Med.

[186] Pajares F, Urdan T, Kirshner B (2008). The ones we remember: scholars reflect on teachers who made a difference. Adolescence and education.

[187] Terwee CB, Bot SD, de Boer MR, van der Windt DA, Knol DL, Dekker J (2007). Quality criteria were proposed for measurement properties of health status questionnaires. J Clin Epidemiol.

[188] Pescosolido B, Jensen P, Martin J, Perry B, Olafsdottir S, Fettes D (2008). Public knowledge and assessment of child mental health problems: Finding form the national stigma study – children. J Am Acad Child Adolesc Psychiatry.

[189] Goffman E (1963). Stigma: notes on the management of spoiled identity.

[190] Thornicroft G (2006). Shunned: discrimination against people with mental illness.

[191] Jones EE, Farina A, Hastorf AH, Marcus H, Miller DT, Scott RA (1984). Social stigma: the psychology of marked relationships.

[192] Ajzen I, Fishbein M (1980). Understanding attitudes and predicting social behavior.

