# Supplementary material for: Mental health literacy measures evaluating knowledge, attitudes and help-seeking: a scoping review
Source: BMC Psychiatry. 2015 Nov 17;15:291. doi: 10.1186/s12888-015-0681-9 (PMC4650294; doi:10.1186/s12888-015-0681-9)
Supplement: Additional file 1: — Supplementary files contain an example of search strategies in PubMed, and supplementary references of studies that applied mental health literacy measures but did not provide related psychometrics information. (ZIP 97 kb) [file 12888_2015_681_MOESM1_ESM.zip › Additional 1 file search strategiesR2.docx]

Search strategies in PubMed

| **Concept 1** AND **Concept 2** AND **Concept 3** AND **Concept 4** | | | | |
| --- | --- | --- | --- | --- |
|  | **key Mental health disorders and mental health** | **3 aspects of MHL** | **Assessment tool** | **Study type** |
| OR | "Mental Disorders"[Mesh: noexp] OR “mental health”[Mesh: noexp] | “health education”[tiab] | assessment*[tiab] | Reliability[tiab] |
|  | “Substance-related disorders”[Mesh] OR substance use disorder*[tiab] OR “substance abuse”[tiab] OR “substance misuse”[tiab] OR “substance dependence”[tiab] | “health education”[Mesh] | evaluat*[tiab] | effective*[tiab] |
| OR | anxiety disorder*[tiab] OR “anxiety disorders”[Mesh] OR “generalized anxiety disorder”[tiab] OR “separation anxiety disorder”[tiab] OR “social phobia”[tiab] OR “specific phobia”[tiab] OR “panic disorder”[tiab] OR “posttraumatic stress disorder”[tiab] | “mental health literacy”[tiab] | measur*[tiab] | efficac*[tiab] |
| OR | disruptive behavior disorder*[tiab] OR “attention deficit and disruptive behavior disorders”[Mesh] OR “conduct disorder”[tiab] OR “oppositional defiant disorder”[tiab] | “health knowledge”[tiab] | test*[tiab] | “program evaluation”[Mesh] OR “program evaluation”[tiab] |
| OR | “unipolar depression”[tiab] OR “major depressive disorder”[tiab] OR depression[tiab] OR “depressive disorder”[Mesh] OR “depression”[Mesh] | “health curriculum”[tiab] | scale*[tiab] | Validity[tiab] |
| OR | “attention deficit hyperactivity disorder”[tiab] OR ADHD[tiab] | “mental health awareness”[tiab] | assessment tool*[tiab] |  |
|  |  | awareness[Mesh] | psychometrics[Mesh] OR psychometrics[tiab] |  |
| OR |  | “attitude to health”[Mesh] | questionnaires[Mesh] OR questionnaire*[tiab] |  |
| OR |  |  | survey*[tiab] |  |
| OR |  | stigma[tiab] |  |  |
| OR |  | discrimination[tiab] |  |  |
|  |  | “help seeking behavior”[tiab] OR “seeking help”[tiab] |  |  |
