# Supplementary material for: Mental health literacy measures evaluating knowledge, attitudes and help-seeking: a scoping review
Source: BMC Psychiatry. 2015 Nov 17;15:291. doi: 10.1186/s12888-015-0681-9 (PMC4650294; doi:10.1186/s12888-015-0681-9)
Supplement: Additional file 1: — Supplementary files contain an example of search strategies in PubMed, and supplementary references of studies that applied mental health literacy measures but did not provide related psychometrics information. (ZIP 97 kb) [file 12888_2015_681_MOESM1_ESM.zip › Additional file 2 supplementary references BMC psychiatryR2.docx]

Supplementary references:

1. Abraham A, Easow JM, Ravichandren P, Mushtaq S, Butterworth L, Luty J. Effectiveness and confusion of the Time to Change anti-stigma campaign. The Psychiatrist. 2010; 34: 230-3. doi: 10.1192/pb.bp.109.027052.
2. Adewuya AO, Makanjuola RO. Social distance towards people with mental illness amongst nigerian university students. Social Psychiatry and Psychiatric Epidemiology. 2005; 40(11): 865-8. doi:10.1007/s00127-005-0965-3.
3. Aho-Mustonen K, Tiihonen J, Repo-Tiihonen E, Ryynänen O, Miettinen R, Räty H. Group psychoeducation for long-term offender patients with schizophrenia: An exploratory randomised controlled trial. Criminal Behaviour and Mental Health. 2011; 21(3):163-76.  <http://ezproxy.library.dal.ca/login?url=http://search.ebscohost.com/login.aspx?direct=true&db=psyh&AN=2012-11033-002&site=ehost-live>. Accessed 14 Aug 2015.
4. Aho-Mustonen K, Miettinen R, Koivisto H, Timonen T, Räty H. Group psychoeducation for forensic and dangerous non-forensic long-term patients with schizophrenia: a pilot study. Eur. J. Psychiat. 2008; 22(2): 84-92.
5. Armstrong G, Kermode M, Raja S, Suja S, Chandra P, Jorm AF. A mental health training program for community health workers in india: Impact on knowledge and attitudes. International Journal of Mental Health Systems. 2011; 5(1):17. doi:10.1186/1752-4458-5-17; 10.1186/1752-4458-5-17.
6. Aromaa E, Tolvanen A, Tuulari J, Wahlbeck K. Predictors of stigmatizing attitudes towards people with mental disorders in a general population in Finland. Nordic Journal of Psychiatry. 2011; 65(2): 125-32. doi:10.3109/08039488.2010.510206; 10.3109/08039488.2010.510206.
7. Ay P, Save D, Fidanoglu O. Does stigma concerning mental disorders differ through medical education? A survey among medical students in Istanbul. Social Psychiatry and Psychiatric Epidemiology. 2006; 41: 63-7.
8. Bapat S, Jorm A, Lawrence K. Evaluation of a mental health literacy training program for junior sporting clubs. Australasian Psychiatry: Bulletin of Royal Australian and New Zealand College of Psychiatrists. 2009; 17(6): 475-9. doi:10.1080/10398560902964586.
9. Barney S, Corser GC, White LH. Service-learning with the mentally ill. Michigan Journal of Community Service Learning. 2010; 66-77.
10. Barry M. Community perceptions of mental disorder: an Irish perspective. The Irish Journal of Psychology. 1994; 15(2 & 3): 418-41.
11. Batterham PJ, Griffiths KM, Barney LJ, Parsons A. Predictors of generalized anxiety disorder stigma. Psychiatry Research. 2013; 206(2-3): 282-6. doi:10.1016/j.psychres.2012.11.018; 10.1016/j.psychres.2012.11.018.
12. Bayar MR, Poyraz BC, Aksoy-Poyraz C, Arikan MK. Reducing mental illness stigma in mental health professionals using a web-based approach. The Israel Journal of Psychiatry and Related Sciences. 2009; 46(3): 226-30.
13. Beckman L. Locus of control and attitudes toward mental illness among mental health volunteers. Journal of Consulting and Clinical Psychology. 1972; 38(1): 84-9.
14. Bentz WK, Edgerton JW, Miller FT. Attitudes of teachers and the public toward mental illness. Mental Hygiene. 1971; 55(3): 324-30.
15. Berkowitz R, Eberlein-Fries R, Kuipers L, Leff J. Educating relatives about schizophrenia. Schizophrenia Bulletin. 1984; 10(3): 418-29.
16. Birchwood M, Smith J, Cochrane R. Specific and non-specific effects of educational intervention for families living with schizophrenia. A comparison of three methods. The British Journal Psychiatry. 1992; 160: 806-14. doi: 10.1192/bjp.160.6.806.
17. Blenkiron P. Coping with depression: a pilot study to assess the efficacy of a self-help audio cassette. British Journal of General Practices. 2001; 466: 366-70.
18. Boysen GA, Vogel DL. Education and mental health stigma: The effects of attribution, biased assimilation, and attitude polarization. Journal of Social and Clinical Psychology. 2008; 27(5): 447-70. doi:10.1521/jscp.2008.27.5.447.
19. Brockington IF, Hall P, Levings J, Murphy C. The community’s tolerance of the mentally ill. The British Journal of Psychiatry. 1993; 162: 93-99. doi: 10.1192/bjp.162.1.93.
20. Brown SA. Implementing a brief hallucination simulation as a mental illness stigma reduction strategy. Community Mental Health Journal. 2010; 46(5): 500-4. doi:10.1007/s10597-009-9229-0.
21. Brown, S. A., Evans, Y., Espenschade, K., & O’Connor, M. (2010). An examination of two brief stigma reduction strategies: Filmed personal contact and hallucination simulations. *Community Mental Health Journal, 46*(5), 494-499. doi:10.1007/s10597-010-9309-1
22. Burns JR, Rapee RM. Adolescent mental health literacy: young people’s knowledge of depression and help seeking. Journal of Adolescence. 2006; 29: 225-39. doi:10.1016/j.adolescence.2005.05.004.
23. Cabassa LJ, Zayas LH. Latino immigrants’ intentions to seek depression care. American Journal of Orthopsychiatry. 2007; 77(2): 231-42. doi: 10.1037/0002-9432.77.2.231.
24. Campbell M, Shryane N, Byrne R, Morrison AP. A mental health promotion approach to reducing discrimination about psychosis in teenagers. Psychosis. 2011; 3(1): 41-51. doi:10.1080/17522431003735529.
25. Caporino NE, Chen JI, Karver MS. Preliminary examination of ethnic group differences in adolescent girls' attitudes toward depression treatments. Cultural

Diversity and Ethnic Minority Psychology. 2013; doi:10.1037/a0033467.

1. Carlton PA, Deane FP. Impact of attitudes and suicidal ideation on adolescents' intentions to seek professional psychological help. Journal of Adolescence. 2000; 23: 35-45. doi: 10.1006/jado.1999.0299.
2. Cates ME, Burton AR, Woolley TW. Attitudes of pharmacists toward mental illness and providing pharmaceutical care to the mentally ill. The [Annals of Pharmacotherapy](http://www.google.ca/url?sa=t&rct=j&q=&esrc=s&source=web&cd=1&cad=rja&uact=8&ved=0CCEQFjAAahUKEwjM26OA7e_GAhUCXh4KHbiOAGI&url=http%3A%2F%2Faop.sagepub.com%2F&ei=0SGwVYyvJoK8ebidgpAG&usg=AFQjCNGwIgP2FOqqzuFIDO4tI3R4W13oaw&bvm=bv.98197061,d.dmo). 2005; 39: 1450-5. doi:10.1345/aph.1G009.
3. Chamberlain PN, Goldney RD, Taylor AW, Kerena A. Eckert KA. Have Mental Health Education Programs Influenced the Mental Health Literacy of Those with Major Depression and Suicidal Ideation? A Comparison between 1998 and 2008 in South Australia. Suicide and Life-Threatening Behavior. 2012; 42(5): 525-40. doi: 10.1111/j.1943-278X.2012.00109.x.
4. Chan JYN, Mak WWS, Law LSC. Combining education and video-based contact to reduce stigma of mental illness: ‘‘The Same or Not the Same’’ anti-stigma program for secondary schools in Hong Kong. Social Science & Medicine. 2009; 68: 1521–6. doi:10.1016/j.socscimed.2009.02.016.
5. Chung KF, Chen EYH, Liu CSM. University students' attitudes towards mental patients and psychiatric treatment. International Journal of Social Psychiatry. 2001; 47: 63. doi:10.1177/002076400104700206.
6. Clement S, van Nieuwenhuizen A, Kassam A, Flach C, Lazarus A, de Castro M, et al. Filmed v. live social contact interventions to reduce stigma: randomised controlled trial. The British Journal of Psychiatry. 2012; 201: 57–64. doi: 10.1192/bjp.bp.111.093120.
7. Cohen J, Struening E. Opinions about mental illness: hospital differences in attitude for eight occupation groups. Psychological Reports. 1965; 17: 25-26.
8. Cohen D. The effectiveness of videotape in patient education on depression. The Journal of Biocommunication. 1983; 10(1): 19-23.
9. Conrad I, Dietrich S, Heider D, Blume A, Angermeyer MC, Riedel-Heller S. (2009). 'Crazy? so what!': A school programme to promote mental health and reduce stigma—Results of a pilot study. Health Education. 2009; 109(4): 314-28. doi:10.1108/09654280910970893.
10. Constantine M. Predictors of satisfaction with counseling: racial and ethnic minority clients’ attitudes toward counseling and ratings of their counselors’ general and multicultural counseling competence. Journal of Counseling Psychology. 2002; 49(2): 255–63. doi: 10.1037//0022-0167.49.2.255.
11. Cook TM, Wang J. Descriptive epidemiology of stigma against depression in a general population sample in Alberta. BMC Psychiatry. 2010; 10: 29-244X-10-29. doi:10.1186/1471-244X-10-29; 10.1186/1471-244X-10-29.
12. Corrigan PW, Edwards AB, Qreen A, Diwan SL, Penn DL. Prejudice, social distance, and familiarity with mental illness. Schizophrenia Bulletin. 2001; 27(2): 219-25.
13. Corrigan PW, Watson AC, Warpinski AC, Gracia G. Stigmatizing attitudes about mental illness and allocation of resources to mental health services. Community Mental Health Journal. 2004; 40(4): 297-307.
14. Corrigan PW, Watson AC, Otey E, Westbrook AL, Gardner AL, Lamb TA. How do children stigmatizing people with mental illness? Journal of Applied Social Psychology. 2007; 37(7): 1405-17.
15. Corrigan PW, Rafacz JD, Hautamaki J, Walton J, Rusch N, Rao D, et al. Changing stigmatizing perceptions and recollections about mental illness: the effects of NAMI’s In Our Own Voice. Community Mental Health Journal. 2010; 46: 517-22. doi: 10.1007/s10597-009-9287-3.
16. Corrigan PW, Sokol KA, Rusch N. The impact of self-stigma and mutual help programs on the quality of life of people with serious mental illnesses. Community Mental Health Journal. 2013; 49: 1-6. Doi: 10.1007/s10597-011-9445-2.
17. Costantine MG. Predictors of satisfaction with counseling: racial and ethnic minority clients’ attitudes toward counseling and ratings of their counselors’ general and multicultural counseling competence. Journal of Counseling Psychology. 2002; 49(2): 255-63. doi: 10.1037//0022-0167.49.2.255.
18. Cotton D. The attitudes of Canadian police officers toward the mentally ill. International Journal of Law and Psychiatry. 2004; 27: 135-46. doi:10.1016/j.ijlp.2004.01.004.
19. Couture SM, Penn DL. The effects of prospective naturalistic contact on the stigma of mental illness. Journal of Community Psychology. 2006; 34(5): 635-45. doi: 10.1002/jcop.20119.
20. Covarrubias I, Han M. Mental health stigma about serious mental illness among MSW students: Social contact and attitude. Social Work. 2011; 56(4): 317-25.
21. Creech SK. Changes in attitudes about mental illness among nursing students following a psychiatric affiliation. Journal of Psychiatric Nursing and Mental Health Services. 1977; 15(6): 9-14.
22. Crisp AH, Gelder MG, Rix S, Meltzer HI, Rowlands OJ. Stigmatisation of people with mental illnesses. The British Journal of Psychiatry. 2000; 177: 4-7. doi: 10.1192/bjp.177.1.4.
23. Crisp A, Gelder M, Goddard E, Meltzer H. Stigmatization of people with mental illnesses: a follow-up study within the Changing Minds campaign of the royal college of psychiatrists. World Psychiatry. 2005; 4(2): 106-13.
24. De Jong MA, Mather J. Undergraduate students’ perceptions of schizophrenia. International Journal of Mental Health Addiction. 2009; 7: 458-67. doi: 10.1007/s11469-009-9197-1.
25. Demyan AL, Anderson T. Effects of a brief media intervention on expectations, attitudes, and intentions of mental health help seeking. Journal of Counseling Psychology. 2012; 59(2): 222-9. doi:10.1037/a0026541; 10.1037/a0026541.
26. DeSocio J, Stember L, Schrinsky J. Teaching children about mental health and illness: A school nurse health education program. The Journal of School Nursing. 2006; 22(2): 81-6. doi:10.1177/105984050602200204.
27. Dietrich S, Mergl R, Freudenberg P, Althaus D, Ulrich Heger U. Impact of a campaign on the public’s attitudes towards depression. Health Education Research. 2010; 25(1): 135-50. doi:10.1093/her/cyp050.
28. Drolen CS. The effects of educational setting on student opinions of mental illness. Community Mental Health Journal. 1993; 29(3): 223-34.
29. Dunn KI, Goldney RD, Grande ED, Taylor A. Quantification and examination of depression-related mental health literacy. Journal of Evaluation in Clinical Practice. 2009; 15(4): 650-3. doi:10.1111/j.1365-2753.2008.01067.x; 10.1111/j.1365-2753.2008.01067.x.
30. Durand-Zaleski I, Scott J, Rouillon F, Marion Leboyer M. A first national survey of knowledge, attitudes and behaviours towards schizophrenia, bipolar disorders and autism in France. BMC Psychiatry. 2012; 12: 128. <http://www.biomedcentral.com/1471-244X/12/128>. Accessed 14 Aug 2015.
31. Ebneter DS, Dipl Psych D, Latner JD. Stigmatizing attitudes differ across mental health disorders: a comparison of stigma across eating disorders, obesity, and major depressive disorder. Journal of Nervous and Mental Disease. 2013; 201: 281-5. doi: 10.1097/NMD.0b013e318288e23f.
32. Economou M, Louki E, Peppou LE, Gramandani C, Yotis L, Stefanis CN. Fighting psychiatric stigma in the classroom: The impact of an educational intervention on secondary school students' attitudes to schizophrenia. The International Journal of Social Psychiatry. 2012; 58(5): 544-51. doi:10.1177/0020764011413678; 10.1177/0020764011413678.
33. Ertugrul A, Berna Ulug B. Perception of stigma among patients with schizophrenia. Social Psychiatry and Psychiatric Epidemiology. 2003; 39: 73-7. doi: 10.1007/s00127-004-0697-9.
34. Essler V, Arthur A, Stickley T. Using a school-based intervention to challenge stigmatizing attitudes and promote mental health in teenagers. Journal of Mental Health. 2006; 15(2): 243-50. doi:10.1080/09638230600608669.
35. Esters IG, Cooker PG, Ittenbach RF. Effects of a unit of instruction in mental health on rural adolescents' conceptions of mental illness and attitudes about seeking help. Adolescence. 1998; 33(130): 469-76.
36. Evans-Lacko S, London J, Little K, Henderson C, Thornicroft G. Evaluation of a brief anti-stigma campaign in cambridge: Do short-term campaigns work? BMC Public Health. 2010; 10: 339-2458-10-339. doi:10.1186/1471-2458-10-339; 10.1186/1471-2458-10-339.
37. Evans-Lacko S, Brohan E, Mojtabai R, Thornicroft G. Association between public views of mental illness and self-stigma among individuals with mental illness in 14 european countries. Psychological Medicine. 2012; 42(8): 1741-52. doi:10.1017/S0033291711002558; 10.1017/S0033291711002558.
38. Evans-Lacko S, Malcolm E, West K, Rose D, London J, Rüsch, N, et al. Influence of time to Change’s social marketing interventions on stigma in england 2009–2011. The British Journal of Psychiatry, 2013; 202 Suppl: 77-88. doi:10.1192/bjp.bp.113.126672.
39. Finkelstein J, Lapshin O, Evgeny Wasserman E. Randomized study of different anti-stigma media. Patient Education and Counseling. 2008; 71: 204–14. doi:10.1016/j.pec.2008.01.002.
40. Finkelstein J, Lapshin O. Reducing depression stigma using a web-based program. International Journal of Medical Informatics, 2007; 76: 726-34. doi:10.1016/j.ijmedinf.2006.07.004.
41. Fiorillo A, Bassi M, de Girolamo G, Catapano F, Romeo F. The impact of a psychoeducational intervention on family members' views about schizophrenia: Results from the OASIS italian multi-centre study. The International Journal of Social Psychiatry. 2011; 57(6): 596-603. doi:10.1177/0020764010376607; 10.1177/0020764010376607.
42. Fung KMT, Tsang HWH, Cheung W. Randomized controlled trial of the self-stigma reduction program among individuals with schizophrenia. Psychiatry Research. 2011; 189(2): 208-14. doi:10.1016/j.psychres.2011.02.013.
43. Gaebel W, Harald Zäske H, Anja E, Baumann AE, Klosterkötter J, Maier W, Decker P, Möller H. Evaluation of the German WPA “Program against stigma and discrimination because of schizophrenia — Open the Doors”: Results from representative telephone surveys before and after three years of antistigma interventions. Schizophrenia Research. 2008; 98: 184–93. doi:10.1016/j.schres.2007.09.013.
44. Galletly C, Burton C. Improving medical student attitudes towards people with schizophrenia. The Australian and New Zealand Journal of Psychiatry. 2011; 45(6): 473-76. doi:10.3109/00048674.2011.541419; 10.3109/00048674.2011.541419.
45. Gamble C, Midence K, Leff J. The effects of family work training on mental health nurses' attitude to and knowledge of schizophrenia: A replication. Journal of Advanced Nursing. 1994; 19(5): 893-96.
46. Goldney RD, Fisher LJ, Wilson DH, Cheok F. Mental health literacy of those with major depression and suicidal ideation: An impediment to help seeking. Suicide & Life-Threatening Behavior. 2002; 32(4): 394-403.
47. Goldney RD, Fisher LJ, Wilson DH, Cheok F. Mental health literacy of those with major depression and suicidal ideation: An impediment to help seeking. Suicide & Life-Threatening Behavior. 2002; 32(4): 394-403.
48. Goldney RD, Fisher LJ. Have broad-based community and professional education programs influenced mental health literacy and treatment seeking of those with major depression and suicidal ideation? Suicide and Life-Threatening Behavior. 2008; 38(2): 129-42.
49. Coles ME, Heimberg RG, Weiss BD. (2013). The public's knowledge and beliefs about obsessive compulsive disorder. Depression and Anxiety. 2013; 30(8): 778-85. <http://ezproxy.library.dal.ca/login?url=http://search.ebscohost.com/login.aspx?direct=true&db=psyh&AN=2013-27969-012&site=ehost-live>. Accessed 14 Aug 2015.
50. Gonzalez JM, Tinsley HEA, Kreuder KR. Effects of psychoeducational interventions on opinions of mental illness, attitudes toward help seeking, and expectations about psychotherapy in college students. Journal of College Student Development. 2002; 43(1): 51-63.
51. Gonzalez JM, Alegria M, Prihoda TJ. How do attitudes toward mental health treatment vary by age, gender, and ethnicity/race in young adults? Journal of Community Psychology. 2005; 33(5): 611-29. Doi: 10.1002/jcop.20071.
52. Granello DH, Granello PF. Defining mental illness: the relationship between college students’ beliefs about the definition of mental illness and tolerance. Journal of College Counseling. 2000; 3: 100-12.
53. Griffiths KM, Nakane Y, Helen Christensen H,Yoshioka K, Jorm, AF, Nakane H. Stigma in response to mental disorders: a comparison of Australia and Japan*.* BMC Psychiatry. 2006; 6: 21. doi:10.1186/1471-244X-6-21.
54. Hamaideh SH, Mudallal R. Attitudes of Jordanian Nursing Students towards Mental Illness: The Effect of Teaching and Contact on Attitudes Change. College Student Journal. 2009; 43(2): 335-46.
55. Hammer JH, Vogel DL. Men’s help seeking for depression: The efficacy of a male-sensitive brochure about counseling. The Counseling Psychologist. 2010; 38(2): 296-313. doi:10.1177/0011000009351937.
56. Hanzawa S, Bae J, Tanaka H, Tanaka G, Bae Y, Goto M, et al. Family stigma and care burden of schizophrenia patients: comparison between Japan and Korea. Asia-Pacific Psychiatry. 2009; 1(3): 120-129. doi:10.1111/j.1758-5872.2009.00039.x.
57. Hanzawa, S., Nosaki, A., Yatabe, K., Nagai, Y., Tanaka, G., Nakane, H., & Nakane, Y. Study of understanding the internalized stigma of schizophrenia in psychiatric nurses in Japan. Psychiatry and Clinical Neurosciences. 2012; 66(2): 113-20. doi:10.1111/j.1440-1819.2011.02307.x; 10.1111/j.1440-1819.2011.02307.x.
58. Hartman LI, Michel NM, Winter A, Young RE, Flett GL, Goldberg JO. Self-stigma of mental illness in high school youth. Canadian Journal of School Psychology. 2013; 28(1): 28-42. doi:10.1177/0829573512468846.
59. Henderson C, Corker E, Lewis-Holmes E, Hamilton S, Flach C, Rose D, et al. England’s time to change antistigma campaign: One-year outcomes of service user–rated experiences of discrimination. Psychiatric Services. 2012; 63(5): 451-7. doi:10.1176/appi.ps.201100422.
60. Hernan A, Philpot B, Edmonds A, Reddy P. Healthy minds for country youth: Help-seeking for depression among rural adolescents. The Australian Journal of Rural Health. 2010; 18(3): 118-24. doi:10.1111/j.1440-1584.2010.01136.x; 10.1111/j.1440-1584.2010.01136.x.
61. Hernandez MY, Organista KC. Entertainment-education? A fotonovela? A new strategy to improve depression literacy and help-seeking behaviors in at-risk immigrant latinas. American Journal of Community Psychology. 2013; doi:10.1007/s10464-013-9587-1,
62. Hickie IB, Luscombe GM, Davenport TA, Burns JM, Highet NJ. Perspectives of young people on depression: Awareness, experiences, attitudes and treatment preferences. Early Intervention in Psychiatry. 2007; 1(4): 333-9. doi:10.1111/j.1751-7893.2007.00042.x; 10.1111/j.1751-7893.2007.00042.x.
63. Hill K, Startup M. The relationship between internalized stigma, negative symptoms and social functioning in schizophrenia: the mediating role of self-efficacy. Psychiatry Research. 2013; 206: 151–157. <http://dx.doi.org/10.1016/j.psychres.2012.09.056>. Accessed 14 Aug 2015.
64. Holmes EP, Corrigan PW, Williams P, Canar J, Kubiak MA. Changing attitudes about schizophrenia. Schizophrenia Bulletin. 1999; 25(3): 447-56.
65. Hossain D, Gorman D, Eley R. Enhancing the knowledge and skills of advisory and extension agents in mental health issues of farmers. Australasian Psychiatry: Bulletin of Royal Australian and New Zealand College of Psychiatrists, 2009; 17 Suppl 1: 116-20. doi:10.1080/10398560902948365; 10.1080/10398560902948365.
66. Jermain DM, Crismon ML. Students’ attitudes toward the mentally ill before and after clinical rotations. American Journal of Pharmaceutical Education. 1991; 55: 45-8.
67. Jerome L, Gordon M, Hustler P. A comparison of American and Canadian teachers’ knowledge and attitudes towards attention deficit hyperactivity disorder (ADHD). Canadian Journal of Psychiatry. 1994; 39: 563-7.
68. Jorm AF, Christensen H, Medway J, Korten AE, Jacomb PA, Rodgers B. Public belief systems about the helpfulness of interventions for depression: Associations with history of depression and professional help-seeking. Social Psychiatry and Psychiatric Epidemiology. 2000; 35(5): 211-9. doi:10.1007/s001270050230.
69. Jorm AF, Medway J, Christensen H, Korten, AE, Jacomb PA, Rodgers B. Attitudes towards people with depression: Effects on the public's help-seeking and outcome when experiencing common psychiatric symptoms*.*The Australian and New Zealand Journal of Psychiatry. 2000; 34(4): 612-8.
70. Jorm AF, Griffiths KM, Christensen H, Korten AE, Parslow RA, Rodgers B. Providing information about the effectiveness of treatment options to depressed people in the community: A randomized controlled trial of effects on mental health literacy, help-seeking and symptoms. Psychological Medicine. 2003; 33(6): 1071-9.
71. Jorm AF, Kitchener BA, O'Kearney R, Dear KB. Mental health first aid training of the public in a rural area: A cluster randomized trial. BMC Psychiatry. 2004; 4: 33. doi:10.1186/1471-244X-4-33.
72. Jorm AF, Wright A, Morgan AJ. Beliefs about appropriate first aid for young people with mental disorders: Findings from an Australian national survey of youth and parents. Early Intervention in Psychiatry. 2007; 1(1): 61-70. doi:10.1111/j.1751-7893.2007.00012.x; 10.1111/j.1751-7893.2007.00012.x.
73. Jorm AF, Christensen H, Griffiths KM. Changes in depression awareness and attitudes in Australia: the impact of Beyondblue: the national depression initiative. Australian and New Zealand Journal of Psychiatry. 2006; 40(1): 42-6. doi:10.1111/j.1440-1614.2006.01739.x.
74. Jorm AF, Kitchener BA, Sawer MG, Scales H, Cvetkovski S. Mental health first aid training for high school teachers: a cluster randomized trial. BMC Psychiatry. 2010; 10: 51. <http://www.biomedcentral.com/1471-244X/10/51>. Accessed 14 Aug 2015.
75. Jung SH, Kim, HJ. Perceived stigma and quality of life of individuals diagnosed with schizophrenia and receiving psychiatric rehabilitation services: a comparison between the clubhouse model and a rehabilitation skills training model in South Korea. Psychiatric Rehabilitation Journal. 2012; 35(6): 460–5. doi: 10.1037/h0094580.
76. Kapungwe A, Cooper S, Mayeya J, Mwanza J, Mwape L, Sikwese A, et al. Attitudes of primary health care providers towards people with mental illness: Evidence from two districts in Zambia. African Journal of Psychiatry. 2011; 14(4): 290-7. <http://dx.doi.org.ezproxy.library.dal.ca/10.4314/ajpsy.v14i4.6;><http://dx.doi.org.ezproxy.library.dal.ca/10.4314/ajpsy.v14i4.6>. Accessed Aug 14 2015.
77. Kendra MS, Cattaneo LB, Mohr JJ. Teaching abnormal psychology to improve attitudes toward mental illness and help-seeking. Teaching of Psychology. 2012; 39: 57-61. doi: 10.1177/0098628311430315.
78. Kennedy CW, Polivka BJ, Bininger CJ, Sears JR, Voorhees-Murphy S. Evaluating a mental health education program for community health nurses. Journal of Community Health Nursing. 1995; 12(4): 221-8. doi:10.1207/s15327655jchn1204_4.
79. Kerby J, Calton T, Dimambro B, Flood C, Glazebrook C. Anti-stigma films and medical students’ attitudes towards mental illness and psychiatry: randomised controlled trial. Psychiatric Bulletin. 2008; 32: 345-9. doi:10.1192/pb.bp.107.017152.
80. Kermode M, Bowen K, Arole S, Pathare S, Jorm AF. Attitudes to people with mental disorders: A mental health literacy survey in a rural area of Maharashtra, India. Social Psychiatry and Psychiatric Epidemiology. 2009; 44(12): 1087-96. doi:10.1007/s00127-009-0031-7; 10.1007/s00127-009-0031-7.
81. Kitchener BA, Jorm AF. Mental health first aid training for the public: Evaluation of effects on knowledge, attitudes and helping behavior. BMC Psychiatry. 2002; 2: 10.
82. Kitchener BA, Jorm AF. Mental health first aid training in a workplace setting: A randomized controlled trial. BMC Psychiatry. 2004; 4: 23. doi:10.1186/1471-244X-4-23.
83. Kleim B, Vauth R, Adam G, Stieglitz R, Hayward P, Corrigan P. Perceived stigma predicts low self-efficacy and poor coping in schizophrenia. Journal of Mental Health. 2008; 17(5): 482-91. doi:10.1080/09638230701506283.
84. Klimes-Dougan B, Lee CS, Alaa K, Houri AK. Suicide prevention with adolescents: considering potential benefits and untoward effects of public service announcements. Crisis. 2009; 30(3): 128–35. doi: 10.1027/0227-5910.30.3.128.
85. Krajewski C, Burazeri G, Brand H. Self-stigma, perceived discrimination and empowerment among people with a mental illness in six countries: Pan European stigma study. Psychiatry Research. 2013; 210: 1136–46. <http://dx.doi.org/10.1016/j.psychres.2013.08.013>. Accessed 14 Aug 2015.
86. Kurumatani T, Ukawa K, Kawaguchi Y, Miyata S, Suzuki M, Ide H, et al. Teachers' knowledge, beliefs and attitudes concerning schizophrenia- a cross-cultural approach in japan and taiwan. Social Psychiatry and Psychiatric Epidemiology. 2004; 39(5): 402-9. doi:10.1007/s00127-004-0758-0.
87. Kutcher S, Wei Y, McLuckie A, Bullock L. Educator mental health literacy: a program evaluation of the teacher training education on the Mental Health & High School Curriculum Guide. Advances in School Mental Health Promotion. 2013; 10: 1-11.
88. Landeen JL, Seeman MV, Goering P, Streiner D. Schizophrenia: effects of perceived stigma on two dimensions of recovery. Clinical Schizophrenia & Related Psychoses. 2007; 64-8.
89. Lauber C, Nordt C, Sartorius N, Falcato L, Rössler W. Public acceptance of restrictions on mentally ill people. Acta Psychiatrica Scandinavica, 2000; 102 Suppl 407: 26-32*.*
90. Lauber C, Nordt C, Braunschweig C, Rössler W. Do mental health professionals stigmatize their patients? Acta Psychiatrica Scandinavica. 2006; 113: 51-9. doi:10.1111/j.1600-0447.2005.00718.x.
91. Lazowski L, Koller M, Stuart H, Milev R. Stigma and discrimination in people suffering with a mood disorder: a cross-sectional study. Depression Research and Treatment. 2012; doi:10.1155/2012/724848.
92. Lebowitz MS, Ahn W. Combining biomedical accounts of mental disorders with treatability information to reduce mental illness stigma. Psychiatric Services. 2012; 63(5): 496-9. doi:10.1176/appi.ps.201100265.
93. Leong FTL, Zachar P. Gender and opinions about mental illness as predictors of attitudes toward seeking professional psychological help. British Journal of Guidance & Counselling. 1999; 27(1): 123-32.
94. [Li](http://www.ncbi.nlm.nih.gov/pubmed/?term=Li%20TM%5Bauth%5D) TMH, [Chau](http://www.ncbi.nlm.nih.gov/pubmed/?term=Chau%20M%5Bauth%5D) M, [Wong](http://www.ncbi.nlm.nih.gov/pubmed/?term=Wong%20PW%5Bauth%5D) PWW, [Lai](http://www.ncbi.nlm.nih.gov/pubmed/?term=Lai%20ES%5Bauth%5D) ESY, Yip PSF. Evaluation of a web-based social network electronic game in enhancing mental health literacy for young people. Journal of Medical Internet Research. 2013; 15(5): e80. doi:  [10.2196/jmir.2316](http://dx.doi.org/10.2196%2Fjmir.2316).
95. Link BG, Cullen FT. Contact with the mentally ill and perceptions of how dangerous they are. Journal of Health and Social Behavior. 1986; 27(4): 289-302.
96. Link BG, Cullen FT, Struening E, Shrout PE, Dohrenwend BP. A modified labeling theory approach to mental disorders: an empirical assessment. American Sociological Review. 1989; 54(3): 400-23.
97. Link BG, Mirotznik J, Cullen FT. The effectiveness of stigma coping orientations: can negative consequences of mental illness labeling be avoided? Journal of health and Social Behavior. 1991; 32(3): 302-20.
98. Link BG, Struening EL, Rahav M, Phelan JC, Nuttbrock L. On stigma and its consequences: Evidence from a longitudinal study of men with dual diagnoses of mental illness and substance abuse. Journal of Health and Social Behavior. 1997; 38(2): 177-190.
99. Livingston JD, Tugwell A, Korf-Uzan K, Cianfrone M, Coniglio C. Evaluation of a campaign to improve awareness and attitudes of young people towards mental health issues. Social Psychiatry and Psychiatric Epidemiology. 2013; 48(6): 965-73. doi:10.1007/s00127-012-0617-3; 10.1007/s00127-012-0617-3.
100. Iloabachie C, Wells C, Goodwin B, Baldwin M, Vanderplough-Booth K, Gladstone T, et al. Adolescent and parent experiences with a primary care/Internet-based depression prevention intervention (CATCH-IT). General Hospital Psychiatry. 2011; 33: 543–55. doi:10.1016/j.genhosppsych.2011.08.004.
101. Loch AA, Hengartner MP, Guarniero FB, Lawson FL, Wang YP, Gattaz WF, Rossler W. The more information, the more negative stigma towards schizophrenia: Brazilian general population and psychiatrists compared. Psychiatry Research. 2013; 205(3): 185-91. doi:10.1016/j.psychres.2012.11.023; 10.1016/j.psychres.2012.11.023.
102. Loureiro LM, Jorm AF, Mendes AC, Santos JC, Ferreira RO, Pedreiro AT. Mental health literacy about depression: A survey of Portuguese youth. BMC Psychiatry. 2013; 13(1): 129. doi:10.1186/1471-244X-13-129.
103. Loya F, Reddy R, Hinshaw SP. Mental illness stigma as a mediator of differences in Caucasian and south Asian college students’ attitudes toward psychological counseling. Journal of Counseling Psychology. 2010; 57(4): 484–90. doi: 10.1037/a0021113.
104. Luty J, Rao H, Mary S, Arokiadass R, Easow JM, Sarkhel A. The repentant sinner: methods to reduce stigmatized attitudes towards mental illness*.* Psychiatric Bulletin. 2008; 32: 327-332. doi: 10.1192/pb.bp.107.018457.
105. MacInnes DL, Lewis M. The evaluation of a short group programme to reduce self-stigma in people with serious and enduring mental health problems. Journal of Psychiatric and Mental Health Nursing. 2008; 15(1): 59-65. doi:10.1111/j.1365-2850.2007.01212.x; 10.1111/j.1365-2850.2007.01212.x.
106. Mann CE, Himelein MJ. Putting the person back into psychopathology: an intervention to reduce mental illness stigma in the classroom. Social Psychiatry and Psychiatric Epidemiology. 2008; 43: 545-51. doi: 10.1007/s00127-008-0324-2.
107. Mangum PD, Mitchell KM. Attitudes toward the mentally ill and community care among professionals and their students. Community Mental Health Journal. 1973; 9(4): 350-3.
108. Masuda A, Hayes SC, Lillis J, Bunting K, Herbst SA, Fletcher LB. (2009). The relation between psychological flexibility and mental health stigma in acceptance and commitment therapy: A preliminary process investigation. Behavior and Social Issues. 2009; 18(1): 1-16. <http://ezproxy.library.dal.ca/login?url=http://search.ebscohost.com/login.aspx?direct=true&db=psyh&AN=2010-01242-004&site=ehost-live>. Accessed 14 Aug 2015.
109. Masuda A, Latzman RD. Examining associations among factor-analytically derived components of mental health stigma, distress, and psychological flexibility. Personality and Individual Differences. 2011; 51(4): 435-8. doi:10.1016/j.paid.2011.04.008.
110. Masuda A, Price M, Anderson PL, Schmertz SK, Calamaras MR. The role of psychological flexibility in mental health stigma and psychological distress for the stigmatizer. Journal of Social and Clinical Psychology. 2009; 28(10): 1244-62. doi:10.1521/jscp.2009.28.10.1244.
111. Mayo C, Havelock RG, Simpson DL. Attitudes toward mental illness among psychiatric patients and their wives. Journal of Clinical Psychology. 1971; 27(1): 128-32. doi:10.1002/1097-4679(197101)27:1<128::AID-JCLP2270270133>3.0.CO;2-8.
112. Mbanga NI, Niehaus DJH, Mzamo NC, Wessels CJ, Allen A, Emsley RA, Stein DJ. Attitudes towards and beliefs about schizophrenia in xhosa families with affected probands. Curationis. 2002; 69-73.
113. McKinney KG. Initial evaluation of active minds: a student organization dedicated to reducing the stigma of mental illness. Journal of College Student Psychotherapy. 2009; 23(4): 281-301. doi: 10.1080/87568220903167232.
114. McWilliams S, Hill S, Mannion N, Fetherston A, Kinsella A, O'Callaghan E. Schizophrenia: A five-year follow-up of patient outcome following psycho-education for caregivers. European Psychiatry. 2012; 27(1): 56-61. doi:10.1016/j.eurpsy.2010.08.012
115. Mehta N, Kassam A, Leese M, Butler G, Thornicroft G. Public attitudes towards people with mental illness in England and Scotland, 1994-2003. The British Journal of Psychiatry: The Journal of Mental Science. 2009; 194(3): 278-84. doi:10.1192/bjp.bp.108.052654; 10.1192/bjp.bp.108.052654.
116. Merritt RK, Price JR, Mollison J, Geddes JR. A cluster randomized controlled trial to assess the effectiveness of an intervention to educate students about depression. Psychological Medicine. 2007; 37: 363–72. doi:10.1017/S0033291706009056.
117. Miller MJ, Yang M, Hui K, Choi NY, Lim RH. (2011). Acculturation, enculturation, and Asian American college students' mental health and attitudes toward seeking professional psychological help. Journal of Counseling Psychology. 2011; 58(3): 346-57. doi:10.1037/a0023636; 10.1037/a0023636.
118. Minas H, Colucci E, Jorm AF. Evaluation of mental health first aid training with members of the Vietnamese community in Melbourne, Australia. International Journal of Mental Health Systems. 2009; 3(1): 19-4458-3-19. doi:10.1186/1752-4458-3-19; 10.1186/1752-4458-3-19.
119. Mojtabai R. Americans' attitudes toward mental health treatment seeking: 1990-2003. Psychiatric Services (Washington, D.C.). 2007; 58(5): 642-51. doi:10.1176/appi.ps.58.5.642.
120. Morawska A, Fletcher R, Pope S, Heathwood E, Anderson E, McAuliffe C. Evaluation of mental health first aid training in a diverse community setting. International Journal of Mental Health Nursing. 2013; 22(1): 85-92. doi:10.1111/j.1447-0349.2012.00844.x; 10.1111/j.1447-0349.2012.00844.x.
121. Moses T. Adolescent mental health consumers’ self-stigma: associations with parents’ and adolescents’ illness perceptions and parental stigma. Journal of Community Psychology. 2010; 38(6): 781-98. doi: 10.1002/jcop.20395.
122. Mukherjee R, Fialho A, Wijetunge A, Checinski K, Surgenor T. The stigmatization of psychiatric illness: the attitudes of medical students and doctors in a London teaching hospital. Psychiatric Bulletin. 2002; 26: 178-181. doi: 10.1192/pb.26.5.178.
123. Napoletano MA. The effects of academic instruction in psychology on student nurses’ attitudes toward mental illness. Teaching of Psychology. 1981; 8(1): 22-4.
124. Naylor PB, Cowie HA, Walters SJ, Talamelli L, Dawkins J. Impact of a mental health teaching programme on adolescents. The British Journal of Psychiatry: The Journal of Mental Science. 2009; 194(4): 365-70. doi:10.1192/bjp.bp.108.053058; 10.1192/bjp.bp.108.053058.
125. Ng P, Chan KF. (2002). Attitudes towards people with mental illness: effects of a training program for secondary school students. International Journal of Adolescent Medicine and Health. 2002; 14(3): 215-24.
126. Nguyen E, Chen TF, O'Reilly CL. Evaluating the impact of direct and indirect contact on the mental health stigma of pharmacy students. Social Psychiatry and Psychiatric Epidemiology. 2012; 47(7): 1087-98. doi:10.1007/s00127-011-0413-5; 10.1007/s00127-011-0413-5.
127. Nordt C, Rossler W, Lauber C. Attitudes of mental health professionals toward people with schizophrenia and major depression. Schizophrenia Bulletin. 2006; 32( 4): 709–14. doi:10.1093/schbul/sbj065.
128. Obasi EM, Leong FTL. Psychological distress, acculturation, and mental health-seeking attitudes among people of african descent in the united states: A preliminary investigation. Journal of Counseling Psychology. 2009; 56(2): 227-38. doi:10.1037/a0014865.
129. O'Driscoll C, Heary C, Hennessy E, McKeague L. Explicit and implicit stigma towards peers with mental health problems in childhood and adolescence. Journal of Child Psychology and Psychiatry, and Allied Disciplines. 2012; 53(10): 1054-62. doi:10.1111/j.1469-7610.2012.02580.x; 10.1111/j.1469-7610.2012.02580.x.
130. O’Kearney R, Gibson M, Christensen H, Griffiths KM. Effects of a cognitive-behavioural internet program on depression, vulnerability to depression and stigma in adolescent males: a school-based controlled trial. Cognitive Behaviour Therapy. 2006; 35(1): 43–54.
131. Omizo MM, Kim BSK, Abel NR. Asian and European American cultural values, bicultural competence, and attitudes toward seeking professional psychological help among Asian American adolescents. Journal of Multicutural Counseling and Development. 2008; 36: 15-28.
132. Patten SB, Remillard A, Phillips L, Modgill G, Szeto ACH, Kassam A, Gardner DM. Effectiveness of contact-based education for reducing mental illness-related stigma in pharmacy students. BMC Medical Education. 2012; 12:120. <http://www.biomedcentral.com/1472-6920/12/120>. Accessed 14 Aug 2015.
133. Paykel ES, Hart D, Priest RG. Changes in public attitudes to depression during the Defeat Depression Campaign. The British Journal of Psychiatry. 1998; 73: 519-22. doi: 10.1192/bjp.173.6.519.
134. Payne F, Harvey K, Jessopp L, Plummer S, Tylee A, Gournay K. (2002). Knowledge, confidence and attitudes towards mental health of nurses working in NHS direct and the effects of training. Journal of Advanced Nursing. 2002; 40(5): 549-59.
135. Peluso ETP, Blay SL. Public stigma and schizophrenia in São Paulo city. Revista Brasileira de Psiquiatria. 2011; 33(2): 130-6.
136. Penn DL, Chamberlin C, Mueser KT. The effects of a documentary film about schizophrenia on psychiatric stigma. Schizophrenia Bulletin. 2003; 29(2): 383-91.
137. Penn DL, Link B. Dispelling the stigma of schizophrenia, III: the role of target gender, laboratory-induced contact, and factual information. Psychiatric Rehabilitation Skills. 2002; 6(2): 255-70. doi:10.1080/10973430208408435.
138. Penny NH, Kasar J, Sinay T. Student attitudes toward persons with mental illness: the influence of course work and level I fieldwork. The American Journal of Occupational Therapy. 2002 55(2): 217-20.
139. Penny NH. Longitudinal Study of Student Attitudes Toward People with Mental Illness. Occupational Therapy in Mental Health. 2002; 17(2): 49-80. doi: 10.1300/J004v17n02_04.
140. Perlick DA, Rosenheck RA, Clarkin JF, Sirey JA, Salahi J, Struening EL, Bruce G, Link BG. (2001). Adverse effects of perceived stigma on social adaptation of persons diagnosed with bipolar affective disorder. Psychiatric Services. 2001; 52(12): 1627-32.
141. Perlick DA, Miklowitz DJ, Link BG, Struening E, Kaczynski R, Gonzalez J, et al. Perceived stigma and depression among caregivers of patients with bipolar disorder. The British Journal of Psychiatry: The Journal of Mental Science. 2007; 190: 535-6. doi:10.1192/bjp.bp.105.020826.
142. Perlick DA, Nelson AH, Mattias K, Selzer J, Kalvin C, Wilber CH, et al. In our own voice–family companion: Reducing self-stigma of family members of persons with serious mental illness. Psychiatric Services. 2011; 62(12): 1456-62. doi:10.1176/appi.ps.001222011.
143. Pescosolido BA, Jack K, Martin JK, Long JS, Medina TR, Phelan JC, Link BG. “A Disease Like Any Other”? A decade of change in public reactions to schizophrenia, depression, and alcohol dependence. American Journal of Psychiatry. 2010; 167: 1321–30.
144. Petchers MK, Biegel DE, Drescher R. A video-based program to educate high school students about serious mental illness. Hospital and Community Psychiatry. 1988; 39(10): 1102-3.
145. Phokeo V, Sproule B, Raman-Wilms L. Community pharmacists’ attitudes toward and professional interactions with users of psychiatric medication. Psychiatric Services. 1988; 55(12): 1434-6.
146. Pinfold V, Huxley, P, Thornicroft G, Farmer P, Toulmin H, Graham T. Reducing psychiatric stigma and discrimination: Evaluating an educational intervention with the police force in England. Social Psychiatry and Psychiatric Epidemiology. 2003; 38(6): 337-44. <http://ezproxy.library.dal.ca/login?url=http://search.ebscohost.com/login.aspx?direct=true&db=psyh&AN=2003-06135-009&site=ehost-live>. Accessed 14 Aug 2015.
147. Pinfold V, Thornicroft G, Huxley P, Farmer P. (2005). Active ingredients in anti-stigma programmes in mental health. International Review of Psychiatry. 2005; 17(2): 123-31. doi:10.1080/09540260500073638.
148. Pinto-Foltz MD, Logsdon MC, Myers JA. Feasibility, acceptability, and initial efficacy of a knowledge-contact program to reduce mental illness stigma and improve mental health literacy in adolescents. Social Science & Medicine (1982). 2011; 72(12): 2011-9. doi:10.1016/j.socscimed.2011.04.006;10.1016/j.socscimed.2011.04.006.
149. Pitre N, Stewart S, Adams S, Bedard T, Landry S. The use of puppets with elementary school children in reducing stigmatizing attitudes towards mental illness. Journal of Mental Health. 2007; 16(3): 415-29. doi:10.1080/09638230701299160.
150. Pittman JO, Noh S, Coleman D. Evaluating the effectiveness of a consumer delivered anti-stigma program: Replication with graduate-level helping professionals. Psychiatric Rehabilitation Journal. 2010; 33(3): 236-8.
151. Raguram R, Weiss MG, Channabasavanna SM, Devins GM. Stigma, depression, and Somatization in South India. The American Journal of Psychiatry. 1996; 153(8): 1043-9.
152. Raguram R, Raghu TM, Vounatsou P, Weiss MG. Schizophrenia and the cultural epidemiology of stigma in Bangalore, India. Journal of Nervous and Mental Disease. 2004; 192(11): 734-44. doi:10.1097/01.nmd.0000144692.24993.1b.
153. Reinke RR, Corrigan PW, Leonhard C, Lundin RK, Kubiak MA. (2004). Examining two aspects of contact on the stigma of mental illness. Journal of Social and Clinical Psychology. 2004; 23(3): 377-89.
154. Rusch N, Holzer A, Hermann C, Schramm E, Jacob GA, Bohus M, Corrigan PW. Self-stigma in women with borderline personality disorder and women with social phobia. The Journal of Nervous and Mental Disease. 2006; 194(10): 766-73.
155. Reavley NJ, Jorm AF. Public recognition of mental disorders and beliefs about treatment: changes in Australia over 16 years. The British Journal of Psychiatry: The Journal of Mental Science. 2012; 200(5): 419-25. doi:10.1192/bjp.bp.111.104208; 10.1192/bjp.bp.111.104208.
156. Rickwood DJ, Braithwaite VA. Social-psychological factors affecting help-seeking for emotional problems. Social Science & Medicine. 1994; 39(4): 563-72. doi:10.1016/0277-9536(94)90099-X.
157. Rickwood D, Cavanagh S, Curtis L, Sakrouge R. Educating young people about mental health and mental illness: Evaluating a school-based programme. International Journal of Mental Health Promotion. 2004; 6(4): 23-32. doi:10.1080/14623730.2004.9721941.
158. Ritsher JB, Phelan JC. Internalized stigma predicts erosion of morale among psychiatric outpatients. Psychiatry Research. 2004; 129: 257-65. Doi:10.1016/j.psychres.2004.08.003.
159. Roberts G, Somers J, Dawe J, Passy R, Mays C, Carr G, Shiers D, Jo Smith J. On the edge: a drama-based mental health education programme on early psychosis for schools. Early Intervention in Psychiatry. 2007; 1: 168–76. doi:10.1111/j.1751-7893.2007.00025.x.
160. Romer D, Bock M. Reducing the stigma of mental illness among adolescents and young adults: The effects of treatment information. Journal of Health Communication. 2008; 13(8): 742-58. doi:10.1080/10810730802487406.
161. Ruble AE, Leon PJ, Gilley-Hensley L, Hess SG, Swartz KL. Depression knowledge in high school students: Effectiveness of the adolescent depression awareness program. Journal of Affective Disorders. 2013; 150: 1025–30. http://dx.doi.org/10.1016/j.jad.2013.05.033.
162. Rusch LC, KANTER JW, Angelone AF, Ridley RC. The impact of In Our Own Voice on stigma. American Journal of Psychiatric Rehabilitation. 2008; 11(4): 373-89. DOI: 10.1080/15487760802397660.
163. Rusch LC, Kanter JW, Brondino MJ. A comparison of contextual and biomedical models of stigma reduction for depression with a nonclinical undergraduate sample. The Journal of Nervous and Mental Disease. 2009; 197(2): 104-110.
164. Santor DA, Poulin C, LeBlanc JC, Kusumakar V. Facilitating help seeking behavior and referrals for mental health difficulties in school aged boys and girls: a school-based intervention. Journal of Youth and Adolescence. 2007; 36: 741–52. doi: 10.1007/s10964-006-9092-z.
165. Santor DA, Poulin C, LeBlanc JC, Kusumakar V. Online health promotion, early identification of difficulties, and help seeking in young people. Journal of American Academy of Child and Adolescent Psychiatry. 2007; 46(1): 50-59. doi: 10.1097/01.chi.0000242247.45915.ee.
166. Scheyett A, Kim M. “Can We Talk?”: using facilitated dialogue to positively change student attitudes towards persons with mental illness. Journal of Teaching in Social Work. 2004; 24(1/2): 39-54. doi:10.1300/J067v24n01_03.
167. Schmall VL, Pratt CC. Community education on mental health in later life: Findings from an evaluation of a series of three model programs. Journal of Mental Health Administration. 1993; 20(3): 190-200.
168. Schulze B, Richter-Werling M, Matschinger H, Angermeyer MC. Crazy? so what! effects of a school project on students' attitudes towards people with schizophrenia. Acta Psychiatrica Scandinavica. 2003;107(2): 142-50. doi:10.1034/j.1600-0447.2003.02444.x.
169. Sciutto MJ. ADHD knowledge, misconceptions, and treatment acceptability. Journal of Attention Disorders. 2013; 1-8. doi: 10.1177/1087054713493316.
170. Segal SP, Silverman CJ, Temkin TL. Self-stigma and empowerment in combined-CMHA and consumer-run services: Two controlled trials. Psychiatric Services (Washington, D.C.). 2013; doi:10.1176/appi.ps.201200490; 10.1176/appi.ps.201200490.
171. Seo M, Kim HL. Effectiveness of an education program to reduce negative attitudes toward persons with mental illness using online media. Asian Nursing Research. 2010; 4(2): 90–101.
172. Sévigny R, Yang W, Zhang P, Marleau JD, Yang Z, Su L, et al. Attitudes toward the mentally ill in a sample of professionals working in a psychiatric hospital in Beijing. International Journal of Social Psychiatry. 1999; 45: 41. doi: 10.1177/002076409904500106.
173. Sharp W, Hargrove DS, Johnson L, Deal WP. Mental health education: An evaluation of a classroom based strategy to modify help seeking for mental health problems. Journal of College Student Development. 2006; 47(4): 419-38. doi:10.1353/csd.2006.0051.
174. Sheffield JK, Fiorenza E, Sofronoff K. Adolescents’ willingness to seek psychological help: promoting and preventing factors. Journal of Youth and Adolescence. 2004; 33(6): 495–507.
175. Silva de Crane R, Spielberger CD. Attitudes of Hispanic, Black, and Caucasion university students toward mental illness. Hispanic Journal of Behavioral Sciences. 1981; 3: 241-55. doi: 10.1177/073998638100300302.
176. Smith JJ. Psychiatric hospital experience and attitudes toward “mental illness”. Journal of Consulting and Clinical Psychology. 1969; 33(3): 302-6.
177. Smith AL, Cashwell CS. Stigma and mental illness: investigating attitudes of mental health and non-mental-health professionals and trainees. Journal of Humanistic Counselling, Education and Development. 2010; 49: 189-202.
178. Sorsdahl KR, Kakuma R, Wilson Z, Stein DJ. The internalized stigma experienced by members of a mental health advocacy group in South Africa. The International Journal of Social Psychiatry. 2012; 58(1): 55-61. doi:10.1177/0020764010387058; 10.1177/0020764010387058.
179. Sota S, Shimodera S, Kii M, Okamura K, Suto K, Suwaki M, et al. Effect of a family psychoeducational program on relatives of schizophrenia patients. Psychiatry and Clinical Neurosciences. 2008; 62: 379–85. doi:10.1111/j.1440-1819.2008.01815.x.
180. Spagnolo AB, Murphy AA, Librera LA. Reducing stigma by meeting and learning from people with mental illness. Psychiatric Rehabilitation Journal. 2008; 31(3): 186-193. doi:10.2975/31.3.2008.186.193; 10.2975/31.3.2008.186.193.
181. Stanford M, Philpott D. Baptist senior pastors’ knowledge and perceptions of mental illness. Mental Health, Religion & Culture. 2011; 14(3): 281-290. doi:10.1080/13674670903511135.
182. Stansbury KL, Wimsatt M, Simpson GM, Martin F, Nelson N. African American college students: literacy of depression and help seeking. Journal of College Student Development. 2011; 52(4): 497-502. doi: 10.1353/csd.2011.0058.
183. Stone AM, Merlo LJ. Attitudes of college students toward mental illness stigma and the misuse of psychiatric medications. Journal of Clinical Psychiatry. 2011; 72(2): 134-9.
184. Stuart H. Reaching out to high school youth: The effectiveness of a video-based antistigma program. Canadian Journal of Psychiatry.Revue Canadienne De Psychiatrie. 2006; 51(10): 647-53.
185. Stuart H, Arboleda-Florez J. Community attitudes toward people with schizophrenia. Canadian Journal of Psychiatry. 2001; 46: 245-52.
186. Sussman LK, Robins LN, Earls F. Treatment-seeking for depression by black and white Americans. Social Science and Medicine. 1987; 24(3): 187-96.
187. Swartz KL, Kastelic EA, Hess SG, Cox TS, Gonzales LC, Mink SP, DePaulo Jr J R. The effectiveness of a school-based adolescent depression education program. Health Education and Behavior. 2010; 37(1): 11-22. doi: 10.1177/1090198107303313.
188. Tanaka G, Ogawa T, Inadomi H, Kikuchi Y, Ohta Y. Effects of an educational program on public attitudes towards mental illness. Psychiatry and Clinical Neurosciences. 2003; 57(6): 595-602. doi:10.1046/j.1440-1819.2003.01173.x.
189. Theriot MT, Lodato GA. Attitudes about mental illness and professional danger among new social work students. Journal of Social Work Education. 2012; 48(3): 403-23. doi: 10.5175/JSWE.2012.201000079.
190. Thompson AH, Stuart H, Bland RC, Arboleda-Florez J, Warner R, Dickson RA. Attitudes about schizophrenia from the pilot site of the WPA worldwide campaign against the stigma of schizophrenia. Social Psychiatry and Psychiatric Epidemiology. 2002; 37(10): 475-82. doi:10.1007/s00127-002-0583-2.
191. Thornicroft G, Brohan E, Rose D, Sartorius N, Leese M, for the INDIGO Study Group. Global pattern of experienced and anticipated discrimination against people with schizophrenia: a cross-sectional survey. Lancet. 2009; 373(31): 408-15. doi:10.1016/S0140-6736(08)61817-6.
192. Tieu Y, Konnert C, Wang J. Depression literacy among older Chinese immigrants in Canada: A comparison with a population-based survey. International Psychogeriatrics / IPA. 2010; 22(8): 1318-26. doi:10.1017/S1041610210001511; 10.1017/S1041610210001511.
193. Tomaras VD, Ginieri-Coccossis M, Vassiliadou M, Malliori M, Ferentinos S, Soldatos CR, Tylee A. Education in mental health promotion and its impact on the participants' attitudes and perceived mental health. Annals of General Psychiatry. 2011; 10: 33-859X-10-33. doi:10.1186/1744-859X-10-33; 10.1186/1744-859X-10-33.
194. Twardzicki M. Challenging stigma around mental illness and promoting social inclusion using the performing arts. The Journal of the Royal Society for the Promotion of Health. 2008; 128(2): 68-72.
195. Ukpong DI, Abasiubong F. Stigmatising attitudes towards the mentally ill: a survey in a Nigerian university teaching hospital. SAJP. 2010; 16(2): 56-60.
196. Unger JB, Cabassa LJ, Molina GB, Contreras S, Baron M. Evaluation of a fotonovela to increase depression knowledge and reduce stigma among Hispanic adults. Journal of Immigrant and Minority Health / Center for Minority Public Health. 2013; 15(2): 398-406. doi:10.1007/s10903-012-9623-5; 10.1007/s10903-012-9623-5.
197. Van Dorn RA, Swanson JW, Elbogen EB, Swartz MS. A comparison of stigmatizing attitudes toward persons with schizophrenia in four stakeholder groups: perceived likelihood of violence and desire for social distance. Psychiatry. 2005; 68(2): 152-63.
198. Virtala A, Salmelin R, Tamminen T, Anttinen E. (1998). Change of attitudes towards psychiatric issues during medical education. Medical Teacher. 1998; 20(4): 356-63.
199. Vogel DL, Wester SR. To seek help or not to seek help: the risks of self-disclosure. Journal of Counseling Psychology. 2003; 50(3): 351-61. doi:10.1037/0022-0167.50.3.351.
200. Vogel DL, Wester SR. The role of outcome expectations and attitudes on decisions to seek professional help. Journal of Counseling Psychology. 2005; 52(4): 459-70. doi:10.1037/0022-0167.52.4.459.
201. Vogel DL, Bitman RL, Hammer JH, Wade NG. Is stigma internalized? the longitudinal impact of public stigma on self-stigma. Journal of Counseling Psychology. 2013; doi:10.1037/a0031889.
202. Wade NG, Post BC, Cornish MA, Vogel DL, Tucker JR. Predictors of the change in self-stigma following a single session of group counseling. Journal of Counseling Psychology. 2011; 58(2): 170-82. doi: 10.1037/a0022630.
203. Ward EC, Heidrich SM. African American women’s beliefs about mental illness, stigma, and preferred coping behaviors. Research in Nursing & Health. 2009; 32: 480–92. doi: 10.1002/nur.20344.
204. Walker I, Read J. The differential effectiveness of psychosocial and biogenetic causal explanations in reducing negative attitudes toward "mental illness". Psychiatry. 2002; 65(4): 313-25.
205. Watson AC, Otey E, Westbrook AL, Gardner AL, Lamb TA, Corrigan PW, Fenton WS. (2004). Changing middle schoolers' attitudes about mental illness through education. Schizophrenia Bulletin. 2004; 30(3): 563-72. <http://ezproxy.library.dal.ca/login?url=http://search.ebscohost.com/login.aspx?direct=true&db=psyh&AN=2004-21937-007&site=ehost-live>. Accessed 14 Aug 2015.
206. Watson AC, Corrigan P, Larson JE, Sells M. Self-stigma in people with mental illness. Schizophrenia Bulletin. 2007; 33(6): 1312–8.
207. Weiss MG, Jadhav S, Raguram R, Vounatsou P, Littlewood R. Psychiatric stigma across cultures: local validation in Bangalore and London. Anthropology & Medicine. 2001; 8(1): 71-87. doi: 10.1080/13648470120063906.
208. Weller L, Grunes S. Does contact with the mentally ill affect nurses’ attitudes to mental illness? British Journal of Medical Psychology. 1988; 61: 277-84.
209. Whatley CD. Social attitudes toward discharged mental patients. Social Problems. 1958-1959; 313-20.
210. Wilson CJ, Rickwood D, Deane FP. Depressive symptoms and help-seeking intentions in young people. Clinical Psychologist. 2007; 11(3): 98-107. doi:10.1080/13284200701870954.
211. Wilson CJ, Deane FP, Marshall KL, Dalley A. Reducing adolescents’ perceived barriers to treatment and increasing help-seeking intentions: effects of classroom presentations by general practitioners. Journal of Youth and Adolescence. 2008; 37: 1257–69. doi: 10.1007/s10964-007-9225-z.
212. Wolff G, Pathare S, Craig T, Leff J. Community knowledge of mental illness and reaction to mentally ill people. British Journal of Psychiatry. 1996; 168: 191-8. doi: 10.1192/bjp.168.2.191.
213. Wong PWC, Fu KW, Chan KYK, Chan WSC, Liu PMY, Law YW, Yip PSF. Effectiveness of a universal school-based programme for preventing depression in Chinese adolescents: A quasi-experimental pilot study. Journal of Affective Disorders. 2012; 142: 106-14. <http://dx.doi.org/10.1016/j.jad.2012.03.050>. Accessed 14 Aug 2015.
214. Worakul P, Thavichachart N, Lueboonthavatchai P. Effects of psycho-educational program on knowledge and attitude upon schizophrenia of schizophrenic patients’ caregivers. Journal of Medical Association of Thailand. 2007; 90(6): 1199-1204.
215. Wright A, Harris MG, Wiggers JH, Jorm AF, Cotton SM, Susy M Harrigan SM, Hurworth RE, Patrick D McGorry PD. Recognition of depression and psychosis by young Australians and their beliefs about treatment. Medical Journal of Australia. 2005; 18(1): 18-23.
216. Wright A, Jorm AF, Mackinnon AJ. Labeling of mental disorders and stigma in young people*.*Social Science & Medicine (1982). 2011; 73(4): 498-506. doi:10.1016/j.socscimed.2011.06.015; 10.1016/j.socscimed.2011.06.015.
217. Wright A, McGorry PD, Harris MG, Jorm AF, Pennell K. Development and evaluation of a youth mental health community awareness campaign - the compass strategy. BMC Public Health. 2006; 6: 215. doi:10.1186/1471-2458-6-215.
218. Yanos PT, Roe D, West ML, Smith SM, Lysaker PH. Group-based treatment for internalized stigma among persons with severe mental illness: Findings from a randomized controlled trial. Psychological Services. 2012; 9(3): 248-58. doi:10.1037/a0028048.
219. Yap MB, Reavley NJ, Jorm AF. Associations between awareness of beyondblue and mental health literacy in Australian youth: Results from a national survey. Australian and New Zealand Journal of Psychiatry. 2012; 46(6): 541-52. doi: 10.1177/0004867411435288.
